# Automated discovery of experimental designs in super-resolution microscopy with XLuminA

**DOI:** 10.1038/s41467-024-54696-y

**Published:** 2024-12-10

**Authors:** Carla Rodríguez, Sören Arlt, Leonhard Möckl, Mario Krenn

**Affiliations:** 1 Max Planck Institute for the Science of Light, Erlangen, Germany; 2 Friedrich-Alexander-University Erlangen-Nuremberg, Faculty of Sciences, Department of Physics, Erlangen, Germany; 3 Friedrich-Alexander-University Erlangen-Nuremberg, Faculty of Medicine 1/CITABLE, Erlangen, Germany; 4 Deutsches Zentrum Immuntherapie (DZI), Erlangen, Germany

**Keywords:** Optics and photonics, Software

## Abstract

Driven by human ingenuity and creativity, the discovery of super-resolution techniques, which circumvent the classical diffraction limit of light, represent a leap in optical microscopy. However, the vast space encompassing all possible experimental configurations suggests that some powerful concepts and techniques might have not been discovered yet, and might never be with a human-driven direct design approach. Thus, AI-based exploration techniques could provide enormous benefit, by exploring this space in a fast, unbiased way. We introduce XLuminA, an open-source computational framework developed using JAX, a high-performance computing library in Python. XLuminA offers enhanced computational speed enabled by JAX’s accelerated linear algebra compiler (XLA), just-in-time compilation, and its seamlessly integrated automatic vectorization, automatic differentiation capabilities and GPU compatibility. XLuminA demonstrates a speed-up of 4 orders of magnitude compared to well-established numerical optimization methods. We showcase XLuminA’s potential by re-discovering three foundational experiments in advanced microscopy, and identifying an unseen experimental blueprint featuring sub-diffraction imaging capabilities. This work constitutes an important step in AI-driven scientific discovery of new concepts in optics and advanced microscopy.

## Abstract (web summary)

Researchers have developed XLuminA, an AI framework for the automated discovery of super-resolution microscopy techniques. With 10,000x faster optimization than traditional methods, it discovers unexplored designs breaking the diffraction limit.

## Introduction

The space of all possible experimental optical configurations is enormous. For example, if we consider experiments that consist of just 10 optical elements chosen from 5 different components (such as lasers, lenses, phase shifters, beam splitters, and cameras), we already get 10 million possible discrete arrangements. The experimental topology (i.e., how the elements are arranged) will further increase this number greatly. Finally, each of these optical components can have tunable parameters (such as lenses’ focal lengths, laser power, or splitting ratios of beam splitters), which lead to additional high-dimensional continuous parameter space for each of the previously mentioned discrete possibilities. This vast search space contains all experimental designs possible, including those with exceptional properties. So far, researchers have been exploring this space of possibilities guided by experience, intuition, and creativity – and have uncovered countless exciting experimental configurations and technologies. But due to the complexity of this space, it might be that some powerful concepts and techniques have not been discovered so far, and might never be with a human-driven direct design approach. This is where AI-based exploration techniques could provide enormous benefit, by exploring the space in a fast, unbiased way^1,2^.

Optical microscopes, in today’s sense, were invented 300 years ago by Antonj van Leeuwenhoek^3^. Since then, few techniques used in the sciences have seen similarly rapid development and impact on diverse fields, ranging from material sciences all the way to medicine^4–7^. Arguably, optical microscopy is currently most widely used in biological sciences, where precise labeling of imaging targets enables fluorescence microscopy with exquisite sensitivity and specificity^8,9^. In the past two decades, several breakthroughs have broadened the scope of optical microscopy in this area even further. Among them, through the ingenuity and creativity of human researchers, the discovery of super-resolution (SR) methods, which circumvent the classical diffraction limit of light, stands out in particular. Examples of versatile and powerful SR techniques are STED^10^, PALM/F-PALM^11,12^, (d)STORM^13,14^, SIM^15^, and MINFLUX^16^, with considerable impact in biology^17–19^, chemistry^20^ and material sciences^21^ for example. Crucially, the motivation of our work goes far beyond small-scale optimization of already-known optical techniques. Rather, this work sets out to discover novel, experimentally viable concepts for advanced optical microscopy that are at present entirely untapped.

**Fig. 1:**
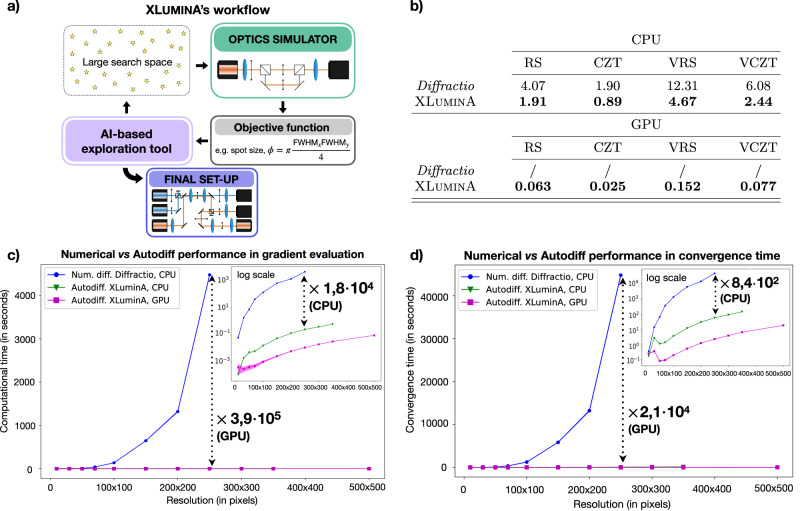
Overview and performance of XLuminA. **a** Software’s workflow shows integrated feedback between the AI discovery tool and the optics simulator. Initial random optical parameters shape the hardware design on a virtual optical table. The optics simulator computes the performance of the experiment through detected light, from which the objective function (for instance, the spot size *ϕ*, where FWHM stands for Full Width Half Maximum) is evaluated. To improve the cost function metric, the optimizer adjusts the optical parameters, creating an iterative cycle between the simulator and optimizer until convergence. **b** Average execution time (in seconds) over 100 runs at 2048 × 2048 pixel resolution, for scalar and vectorial field propagation using Rayleigh-Sommerfeld (RS, VRS) and Chirped z-transform (CZT, VCZT) algorithms in Diffractio and XLuminA. Using pre-compiled jitted functions, XLuminA achieves × 2 speedup for RS and CZT and  × 2.5 for VRS and VCZT on CPU. GPU implementation improves the performance up to × 64 for RS, × 76 for CZT, × 80 for VRS, and × 78 for VCZT. **c** Average time (in seconds) over 5 runs for a single gradient evaluation using numerical differentiation (num. diff) with Diffractio’s optical simulator (blue dots) and auto-differentiation (autodiff) methods (green triangles for CPU and magenta squares for GPU) with XLuminA's optical simulator for different resolutions. At 250 × 250 pixel resolution, GPU-based XLuminA’s autodiff methods significantly outperform numerical methods by a factor of × 3.9 ⋅ 10^5^, and a factor of × 1.8 ⋅ 10^4^ in the CPU. **d** Average time (in seconds) over 5 runs for convergence time, using numerical differentiation with Diffractio’s optical simulator and autodiff methods with XLuminA’s optical simulator for different resolutions. At 250 × 250 pixel resolution, GPU-based XLuminA’s autodiff methods significantly outperform numerical methods by a factor of × 2.1 ⋅ 10^4^ and a factor of × 8.4 ⋅ 10^2^ in the CPU. Standard deviation corresponds to shaded regions. We use BFGS and Adam optimizers, for numerical and autodiff approaches, respectively. The superior efficiency of autodiff over traditional numerical methods allows for highly efficient optimizations, particularly employing the large high resolutions we use (up to 2048 × 2048 pixels).

We introduce XLuminA^22^, an efficient open-source framework developed using JAX^23^, for the ultimate goal of discovering new optical design principles. XLuminA offers enhanced computational speed enabled by its accelerated linear algebra compiler (XLA), just-in-time (jit) compilation, seamlessly integrated automatic vectorization or batching, auto-differentiation capabilities^24^, and GPU compatibility. We leverage its scope with a specific focus on the area of SR microscopy, which is a set of techniques that have revolutionized biological and biomedical research over the past decade, highlighted by the 2014 Chemistry Nobel Prize^25^. The software’s workflow is depicted in Fig. 1a. Fundamentally, the simulator is the heart of digital discovery efforts. It translates an experimental design (one point in the vast space of possible designs) to a physical output. The physical output, such as a detector or camera output, can then be used in an objective function to describe the desired design goal. The simulator can either be called directly by gradient-based optimization techniques, or it can be used for generating the training data for deep-learning-based surrogate models. A simulator that can be used for automated design and discovery of new experimental strategies must be (1) fast, (2) reliable, and (3) general. XLuminA’s optical simulator fulfills precisely the aforementioned requirements for advanced microscopy.

Our approach is radically different from previous strategies that employ AI for the data-driven design of single optical elements^26–28^ or data analysis in microscopy, e.g., denoising, contrast enhancement, or point-spread-function (PSF) engineering^29–32^. While these techniques are influential, they are not meant to change the principle of the experimental approach or the optical layout itself. In contrast, XLuminA is equipped with tools to simulate, optimize, and automatically design new optical setups and concepts from scratch.

In the quantum optics domain, numerous works have recently shown how to automatically design new quantum experiments with advanced computational methods^33–36^, that has led to the discovery of new concepts and numerous blueprints implemented in laboratories^37^. Other simulators such as Strawberry fields focus specifically on optimization in photonic quantum computing^38^.

The field of optical inverse design focuses on the de-novo design of nano-optical components with practical features^39,40^. Examples include on-chip particle accelerators^41^, or wavelength-division multiplexers^42^. The main approach is the development of efficient PDE-solvers for Maxwell’s equations, including efficient ways to compute the gradients of the vast amount of parameters, usually by a physics-inspired technique called the adjoint method^43,44^. These techniques are highly computationally expensive^45^ due to their physical targets. We have different physical targets and thus can apply various different approximations in the beam propagation which significantly speeds up our simulator. Interestingly, the adjoint method can be seen as a special case of auto-differentiation (which we use)^44^.

Several open-source software tools facilitate classical optics phenomena simulations. Some examples are Diffractio for light diffraction and interference simulations^46^, Finesse for simulating gravitational wave detectors^47^, which do not support auto-differentiation nor GPU compatibility; and POPPY, developed as a part of the simulation package of the James Webb Telescope^48^, with GPU compatibility but lacking autodiff capabilities. There are also specialized resources like those focusing on the design of Laguerre-Gaussian mode sorters utilizing multi-plane light conversion (MPLC) methods^49,50^, which also do not support GPU computations and autodiff. While these software solutions offer optics simulation capabilities, XLuminA uniquely integrates simulation with AI-driven automated design powered with JAX’s autodiff, just-in-time compilation, and automatic GPU compatibility.

The paper is structured as follows. We first describe XLuminA and highlight its efficiency and computational speed advantage over conventional approaches. We demonstrate the applicability of our approach by rediscovering three foundational optical layouts. First, using a data-driven learning methodology, we rediscover an optical configuration commonly used to adjust beam and image sizes. Then, following pure AI-exploratory strategies within a fully continuous framework, we rediscover, together with experimental design topologies, a beam-shaping technique as employed in STED (stimulated emission depletion) microscopy^10^ and the SR technique exploiting optical vortices^51^. Ultimately, we showcase XLuminA’s capability for genuine discovery, identifying a previously unreported solution that integrates the underlying physical principles present in the two aforementioned SR techniques into a single experimental blueprint, the performance of which exceeds the capabilities of each individual setup. We then discuss the discovered solutions and the applicability of XLuminA. Finally, we conclude with final remarks and future perspectives.

## Results

In this section, we first describe XLuminA’s workflow and performance. Afterward, we showcase the virtual optical designs generated by XLuminA. As benchmarks, we aim to re-discover three foundational experiments, each one covering different areas in optics. By increasing the complexity of the description of light (from scalar to vectorial field representation), we selected: (1) an optical configuration commonly used to adjust beam and image sizes, (2) beam shaping as employed in STED microscopy^10^, and (3) the super-resolution technique using optical vortices detailed in ref. ^51^. Finally, we demonstrate the discovery of a previously unreported experimental blueprint within a large-scale exploration framework. For the first example, we use a data-driven learning methodology. For the last three, we set up a discovery scheme where no training data is involved. The showcased solutions in both scenarios are the result of running multiple optimizations. Importantly, to ensure simulations that approximate real-world experimental conditions, we have included imperfections, misalignment, and noise sources in all optical components. Therefore, all the results hereby presented are computed considering a wide variety of experimental errors. Further details on the robustness of the framework can be found in the Methods section.

### Software workflow and performance

XLuminA allows for the simulation of classical optics hardware configurations and enables the optimization and automated discovery of unexplored setup designs. The software is developed using JAX^23^, which provides an advantage of enhanced computational speed (enabled by accelerated linear algebra compiler, XLA, with just-in-time compilation, jit) while seamlessly integrating the auto-differentiation framework^24^ and automatic GPU compatibility. It is important to remark that our approach is not restricted to run on CPU (as NumPy-based softwares do): due to JAX-integrated functionalities, by default runs on GPU if available, otherwise automatically falls back to CPU.

The ultimate goal is to discover new concepts and experimental blueprints in optics. Importantly, the most computational expense of an optimization loop comes from running individual optical simulations in each iteration. Thus, it is essential to reduce the computation time by maximizing the speed of optical simulation functions. XLuminA is equipped with an optics simulator that contains a diverse set of optical manipulation, interaction, and measurement technologies. Some specific optical propagation implementations of XLuminA are inspired by the optics framework Diffractio^46^. Diffractio is a high-quality, open-source NumPy-based Python module for optics simulation with an active developer community, and is employed in numerous studies in optics and physics in general. We have rewritten and optimized these optical propagation implementations leveraging JAX’s jit functionality, which allows for highly efficient code execution, although it imposes some restrictions, such as specifying all data structures’ dimensions and ensuring their immutability at compile time. On top of that, we developed specialized functions that significantly expand the software capabilities, such as high-resolution propagation methods and numerous additional optical devices which made the current study possible. Further details on the optics simulator can be found in the Supplementary Information section Features and Performance of XLuminA. We evaluate the performance of our optimized functions against their counterparts in Diffractio. The acquired run times are shown in Fig. 1b. Clearly, our methods significantly enhance computational speeds for simulating light diffraction and propagation. For instance, we observe a speedup of a factor of × 2 for RS (Rayleigh-Sommerfeld, a general Fast Fourier Transform-based light propagation algorithm) and CZT (Chirped z-transform, a speed-up version of RS) and about × 2.5 for VRS and VCZT (the vectorized versions of RS and CZT, respectively) using the CPU. With GPU utilization, the speedup factors are × 64 for RS, × 76 for CZT, × 80 for VRS, and × 78 for VCZT.

To include the automated discovery feature, XLuminA’s optical simulator, and optimizer are tied together by the loss function, as depicted in Fig. 1a. The automated discovery tool is designed to explore the vast parameter space encompassing all possible optical designs. When it comes to the nature of the optimizer, it can be either direct (gradient-based) or deep learning-based (surrogate models or deep generative models, e.g., variational autoencoders^52^). In this work, we adopt a gradient-based strategy, where the experimental setup’s parameters are adjusted iteratively in the steepest descent direction. We first evaluate the time it takes for numerical and analytical (auto-differentiation) methods to compute one gradient evaluation and their convergence times over different resolutions and devices. For this purpose, we use two gradient-descent techniques: the Broyden-Fletcher-Goldfarb-Shanno (BFGS) algorithm^53^, which numerically computes the gradients and higher-order derivative approximations and the Adaptive moment estimation (Adam)^54^, an instance of the stochastic-gradient-descent (SGD) method. While BFGS is part of the open-source SciPy Python library^55^ and operates on the CPU, Adam is integrated within the JAX framework and runs on both CPU and GPU. For this last, we take advantage of JAX’s built-in autodiff framework and compute analytically the gradients of the loss function. Combined with the jit (just-in-time) functionality, this approach enables the optimizer to efficiently construct an internal gradient function, considerably reducing the computational time per iteration. The acquired results are depicted in Fig. 1c, d. A detailed description of both evaluations is provided in the Supplementary Information section Features and Performance of XLuminA. Clearly, autodiff consistently outperforms numerical methods on the gradient evaluation time by up to 4 orders of magnitude on CPU and 5 orders on GPU. In convergence time, autodiff demonstrates superior efficiency up to almost 3 orders of magnitude on CPU and 4 orders on GPU. Given that certain optical elements, such as phase masks, may operate at resolutions as high as 2048 × 2048 pixels, the resulting search space can easily expand to around 8.4 million parameters. This makes the use of autodiff within GPU-accelerated frameworks more appropriate for efficient experimentation. Overall, the computational performance of XLuminA highlights its suitability for running complex simulations and optimizations with a high level of efficiency.

### Data-driven rediscovery

**Fig. 2:**
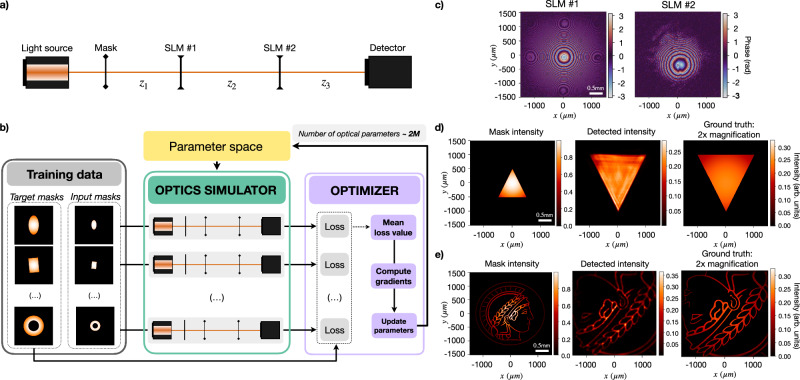
Rediscovery of the optical configuration employed to magnify images. **a** Virtual optical arrangement. It consists of a light source emitting a 650 nm wavelength Gaussian beam. Original lenses are replaced by two spatial light modulators (SLMs) with a resolution of 1024 × 1024 and a pixel size of 2.92 μm. The parameter space (of ∼2 million optical parameters) includes the distances, *z*_1_, *z*_2_, and *z*_3_ (in millimeters) and the phase masks (in radians) of the two SLMs. **b** Data-driven discovery scheme. Input-output sample pairs are fed into the optics simulator in batches of 10. The loss function, computed for each virtual optical setup, evaluates the mean squared error between the intensity response of the system and the corresponding target example from the dataset. The average loss over the batch guides the optical parameter update, which is common to all the virtual optical setups. This cycle is repeated until convergence is reached. **c** Identified phase mask solutions for SLM*#*1 and SLM*#*2. Identified distances correspond to *z*_1_ = 10.14 cm, *z*_2_ = 5.46 cm and *z*_3_ = 7.54 cm. Input, detected intensity, and expected (ground truth) intensity patterns for (**d**) a simple geometry, and (**e**) a complex structure, the Max Planck Society’s logo. In both cases, the identified optical design successfully inverts and magnifies 2 × the input mask.

The optical configuration to adjust beam and image sizes comprises two lenses, each one positioned a focal length apart from their respective input and output planes, *f*_1_ and *f*_2_, respectively, and *f*_1 _+ *f*_2_ from each other. This arrangement performs optical Fourier transformations of input light with magnifications determined by the ratio *f*_2_/*f*_1_. To revisit this design with a magnification of 2 ×, we encoded the virtual setup depicted in Fig. 2a.

The data-driven learning approach is outlined in Fig. 2b. This workflow resembles the dynamics of training, in a supervised way, a physical learning system^56,57^. The system dynamically adjusts the optical elements to effectively transform the input data into the desired configuration. The cost function is computed as

1 $${{\mathcal{L}}}={{\rm{MSE}}}({I}_{{{\rm{Det}}}},{I}_{{{\rm{GT}}}}),$$

where MSE is the mean squared error between the detected intensity pattern from the virtual setup, ${I}_{{{\rm{Det}}}}$ and the corresponding ground truth from the dataset, *I*_GT_. The parameter space comprises 1024 × 1024 pixel phases and 3 optical distances (a total of ∼2 million parameters). Details on the optimization procedure are provided in the Supplementary Information section Data-Driven Rediscovery.

The obtained results, displayed in Fig. 2c, depict lens-like quadratic phases. Notably, the reference model traditionally uses two lenses set at specific distances, yet the identified distances don’t fulfill such a relation. We validate the performance of the identified configuration by imaging the triangle-shaped amplitude mask shown in Fig. 2d, not included in the training data. We further demonstrate how the identified configuration generalizes to complex structures by using the Max Planck Society’s logo in Fig. 2e. In both cases, the detected intensity distribution demonstrates that the optical setup inverts and magnifies the input shape by 2 ×.

### Towards large-scale discovery

**Fig. 3:**
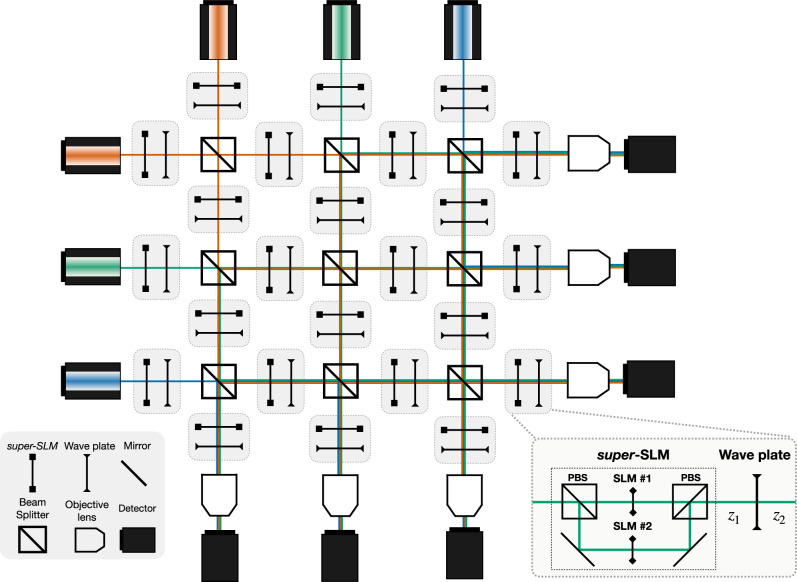
General virtual optical setup for large-scale discovery schemes. Gray boxes represent fundamental building units, each containing a super-SLM and a wave plate positioned a distance *z*_1_ apart. These units are inter-connected through free propagation distances *z*_2_, and beam splitters (PBS). The super-SLM is a hardware box type that consists of two spatial light modulators (SLMs), each one independently imprinting a phase pattern on the horizontal and vertical polarization components of the field. The setup’s complexity and size can be arbitrarily extended by incorporating additional connections, building units, light sources, detectors, etc. The quantization of the large-scale search space generated by this optical setup is provided in the Methods section.

We aim to use XLuminA to discover new microscopy concepts. In essence, discovering new experimental configurations entails a hybrid discrete-continuous search problem. The discrete aspect originates from configuring the optical network topology, whereas the continuous part is linked to the settings of optical elements, such as laser power and beam splitter reflectivity. Discrete-continuous optimization is very difficult computationally, therefore we invent a way to translate this hybrid discrete-continuous optimization problem into a purely continuous optimization problem which can be solved with efficient gradient-based methods. We design the quasi-universal computational ansatz illustrated in Fig. 3, which is designed in a way that setting different (continuous) parameters leads to different optical setup topologies. For a very discrete approach of available parameters, the number of possible discrete arrangements within this general framework scales up to ∼10^20^. Details on this derivation can be found in the Methods section.

Now, the task of XLuminA is to automatically discover experimental design topologies together with their parameter setting, using purely continuous optimization. To achieve this, we initialize the setups with a large and complex optical topology, inspired by other fields that start with highly expressive initial circuits^58,59^. From here, XLuminA should be able to extract much more complex solutions that humans might not have thought about yet^2^.

To test our framework, we target XLuminA to rediscover the concepts of STED microscopy^10^ and the super-resolution (SR) technique using optical vortices^51^. To do so, we conduct an exploration-based optimizing procedure which does not involve training examples. In particular, we first explore the optimization of the optical layout within systems containing pre-defined elements. Later on, we optimize both the optical topology and highly parameterized elements (i.e., SLMs).

### Loss function

The loss function, ${{\mathcal{L}}}$, is calculated as the inverse of the density of the total detected intensity over a certain threshold, *I*_*ε*_. Thus, minimizing ${{\mathcal{L}}}$ aims to maximize the generation of small, high-intensity beams. In particular,

2 $${{\mathcal{L}}}=\frac{1}{{{\rm{Density}}}}=\frac{{{\rm{Area}}}}{{I}_{\varepsilon }}$$

where *I*_*ε*_ is the sum of pixel intensity values greater than the threshold value $\varepsilon \cdot {i}_{\max }$, where 0 ≤ *ε* ≤ 1 and *i*_*max*_ corresponds to the maximum detected intensity. The Area corresponds to the total number of camera pixels fulfilling the same condition. The loss function ${{\mathcal{L}}}$ is common to all the optical setups henceforth described. Importantly, the light gets detected across various devices. Thus, we compute the loss function at each detector and the parameter update is driven by the device demonstrating the minimum loss value. This selection is performed in a fully differentiable manner. Details on the derivation of the loss function are provided in the Methods section.

### Rediscovery through exploration of optical topologies

In this section, we target XLuminA to discover optical topologies for STED^10^ and the sharp focus^51^ techniques within virtual optical tables that contain randomly positioned phase masks displaying fixed patterns. The goal is to discover the optical topology using the available optimizable optical parameters: beam splitter ratios, distances, and wave plate angles. A detailed description of the optimization processes hereby conducted are provided in the Supplementary Information section Rediscovery Through Exploration of Optical Topologies.

STED microscopy^10,60^ is one of the first discovered techniques that circumvent the classical diffraction limit of light. The key idea of this technique is the use of two diffraction-limited laser beams, one probe to activate (excite) the light emitters of the sample and one, doughnut-shaped beam to deactivate its excitation in a controlled way (depletion). Thus, the ultimately detected light is that of the emitters laying in the central region of the doughnut-shaped beam. This effectively reduces the area of normal fluorescence, which leads to super-resolution imaging. To simulate one of the fundamental concepts of STED without having to rely on time-dependent processes, such as the energy level relaxation times of the excited emitters, we perform a nonlinear modulation of the focused light based on the Beer-Lambert law^61^, commonly used to describe the optical attenuation in light-matter interaction. The details of our model are provided in the Supplementary Information section Stimulated Emission Depletion Model.

**Fig. 4:**
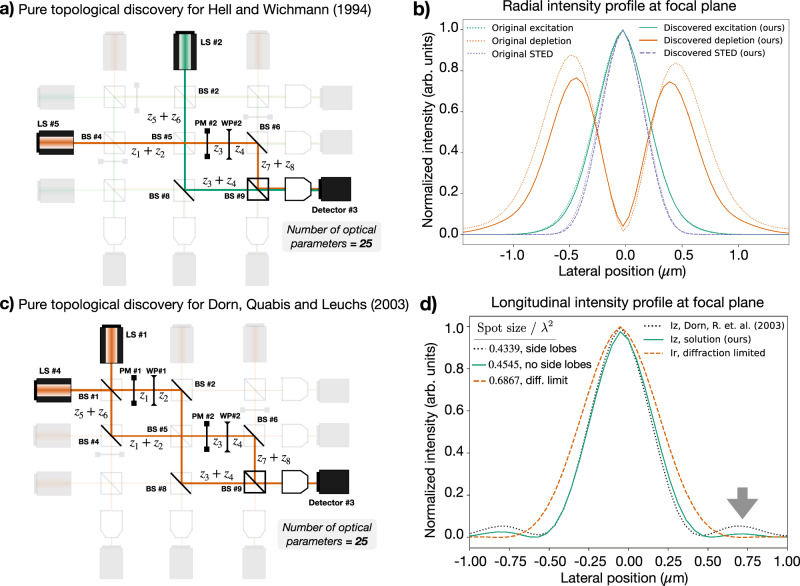
Pure topological discovery within a fully continuous framework. The parameter space (25 optical parameters) is defined by 9 beam splitter ratios (BS), 8 distances (*z*), and 4 wave plates (WP, with variable phase retardance *η* and orientation angle *θ*). **a** Discovered optical topology for STED microscopy^10^. The minimum value of the loss function is demonstrated in detector *#*3. The phase mask (PM) *#*2 corresponds to the radial phase pattern originally used in STED microscopy, which generates a doughnut-shaped beam. **b** Radial intensity profile, ∣*E*_*x*_∣^2^ + ∣*E*_*y*_∣^2^, in horizontal beam section: excitation (green), depletion (orange), and super-resolution effective STED beam (dashed blue line). Lateral position indicates lateral distance from the optical axis. The data corresponding to the original STED phase are indicated with dotted lines. The excitation and depletion beams are diffraction-limited. The effective response breaks the diffraction limit. **c** Discovered optical topology for Dorn, Quabis, and Leuchs (2003)^51^. The minimum value of the loss function is demonstrated in detector *#*3. Phase masks *#*1 and *#*2 correspond to the polarization converter demonstrated in Dorn, Quabis, and Leuchs (2003) and a radial phase pattern originally used in STED microscopy, respectively. Both phase patterns generate, independently, a doughnut-shaped beam. **d** Normalized longitudinal intensity profile, ∣*E*_*z*_∣^2^, for Dorn, Quabis, and Leuchs (2003) and the identified solution (black dotted, and green lines, respectively) and radial intensity profile, ∣*E*_*x*_∣^2^ + ∣*E*_*y*_∣^2^, of the diffraction-limited linearly polarized beam (orange dotted line). Lateral position indicates lateral distance from the optical axis. The spot size is computed as *ϕ* = (*π*/4)FWHM_*x*_ FWHM_*y*_, where FWHM denotes Full Width Half Maximum. The discovered approach breaks the diffraction limit, demonstrating a spot size close to the reference. Also, it does not feature side lobes (indicated with a gray arrow), which can limit practical imaging techniques. Details of the initial optical setup, phase masks, and identified optical parameters can be found in Supplementary Information section Rediscovery Through Exploration of Optical Topologies.

We initialize XLuminA with the virtual optical table in Supplementary Fig. 6a. The loss function corresponds to Equation (2), considering the radial component of the effective beam resulting from the STED process. The discovered topology is depicted in Fig. 4a. It shapes the depletion beam into a doughnut using phase mask *#*2, which corresponds to the spiral phase pattern originally used in STED microscopy. We compute the horizontal cross-section of the focused intensity patterns for both excitation and depletion beams (green and orange lines in Fig. 4b, respectively) and the effective beam (dotted blue line in Fig. 4b). The behavior across the vertical axis yields similar features. For comparison, we also feature the radial intensity profile of the simulated STED reference.

The generation of an ultra-sharp focus is a feature that breaks the diffraction limit in the longitudinal direction, as demonstrated by Dorn, Quabis, and Leuchs in ref. ^51^. This super-resolution is achieved when a radially polarized beam is tightly focused^62,63^. We initialize XLuminA with the virtual optical table in Supplementary Fig. 6b. The loss function corresponds to Equation (2), considering the measured intensity as the field’s longitudinal component. The discovered topology is depicted in Fig. 4c. It shapes the beam into a doughnut using a polarization converter originally used in ref. ^51^ and the spiral phase pattern from STED microscopy (phase masks *#*1 and *#*2 in Fig. 4c, respectively). The longitudinal intensity profiles for the reference and the discovered solution are depicted in Fig. 4d (represented by dotted black and green, respectively). For comparison, we also feature the radial intensity profile of the diffraction-limited beam (dotted orange line in Fig. 4d). The identified solution demonstrates a spot size close to the reference and does not feature side lobes, which can limit practical imaging techniques.

In both cases, we successfully demonstrate how XLuminA can explore different topologies in a fully continuous manner: by adjusting the optical parameters, (e.g., beam splitter ratios), the optimizer can turn off the optical paths.

Importantly, we are not restricted to the use of 3 × 3 optical grids. We conduct an optimization using an optical grid of 6 × 6. The results are presented and discussed in the Supplementary Information section Rediscovery Through Exploration of Optical Topologies.

### Rediscovery through exploration in highly parameterized systems

The results presented thus far predominantly involve optical setups characterized by fixed-phase masks. We further demonstrate XLuminA’s potential in highly parameterized, complex optical systems. We target XLuminA to rediscover the aforementioned SR techniques within the optical table in Supplementary Fig. 8, where super-SLMs are now optimizable. The goal here is to discover both the optical topology and the phase patterns to imprint onto the light beams using the available optimizable optical parameters (i.e., SLMs, distances, beam splitter ratios, and wave plate angles). The optimization processes hereby conducted are detailed in the Supplementary Information section Rediscovery Through Exploration in Highly Parameterized Systems

**Fig. 5:**
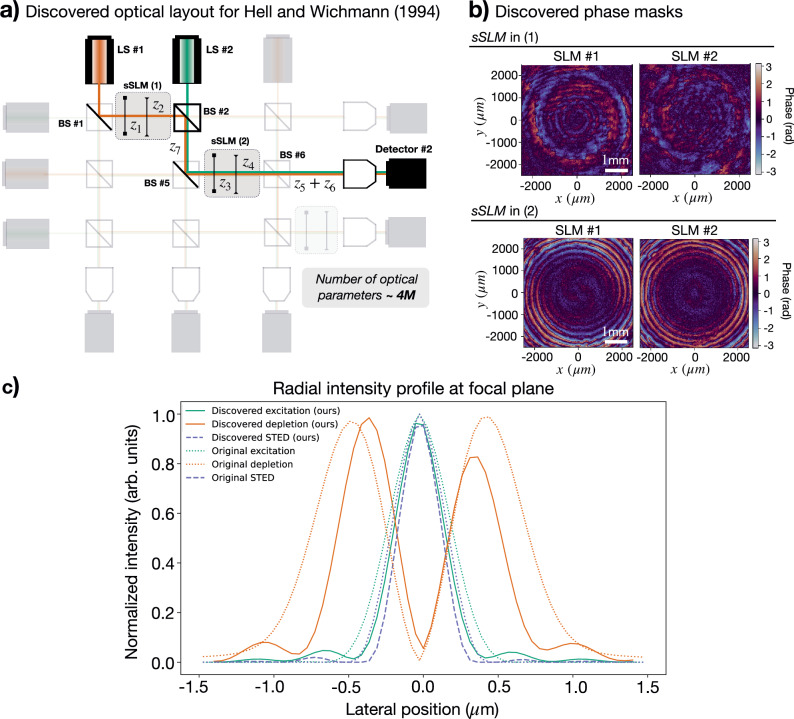
Rediscovery of STED microscopy^10^ within highly parameterized optical systems. **a** Discovered optical topology. The parameter space  (∼4 million optical parameters) is defined by 3 super-SLMs (i.e., 6 SLMs) of (824 × 824) pixel resolution and a computational pixel size of 6.06 μm, 9 beam splitter ratios (BS), 8 distances (*z*) and 3 wave plates (with variable phase retardance *η* and orientation angle *θ*). The minimum value of the loss function is demonstrated in detector *#*2. The setup topology is retrieved from detector *#*2 following the identified beam splitter ratios across the system. The identified optical parameters correspond to: the beam splitter ratios, in [Transmittance, Reflectance] pairs: BS*#*1: [0.000, 0.999], BS*#*2: [0.201, 0.799], BS*#*5: [0.000, 0.999], and BS*#*6: [0.999, 0.000]. The wave plates, in radians (1): *η* = − 1.39, *θ* = − 1.64, and (2): *η* = − 1.61, *θ* = − 0.86. The propagation distances (in cm) are *z*_1_ = 59.52, *z*_2 _= 10.14, *z*_3_ = 76.36, *z*_4_ = 17.93, *z*_5_ = 37.07, *z*_6_ = 65.95, and *z*_7_ = 38.68. **b** Discovered phase patterns for sSLM *#*1 and sSLM *#*2. The speckle-like patterns of SLMs' phase masks are not detrimental to robustness. **c** Radial intensity profile, ∣*E*_*x*_∣^2^ + ∣*E*_*y*_∣^2^, in horizontal beam section: excitation (green), depletion (orange), and super-resolution effective STED beam (dashed blue line). The data corresponding to the original STED experiment - i.e., computed using a spiral phase mask - are indicated with dotted lines. Lateral position indicates lateral distance from the optical axis.

The discovered topology and phase patterns for STED microscopy are depicted in Fig. 5a and b, respectively. As for STED microscopy, the system imprints a phase singularity onto the depletion beam to produce a doughnut shape. In this case, however, it also modulates the excitation beam. The radial intensity profiles of the discovered solution and the reference experiment are depicted in Fig. 5c.

**Fig. 6:**
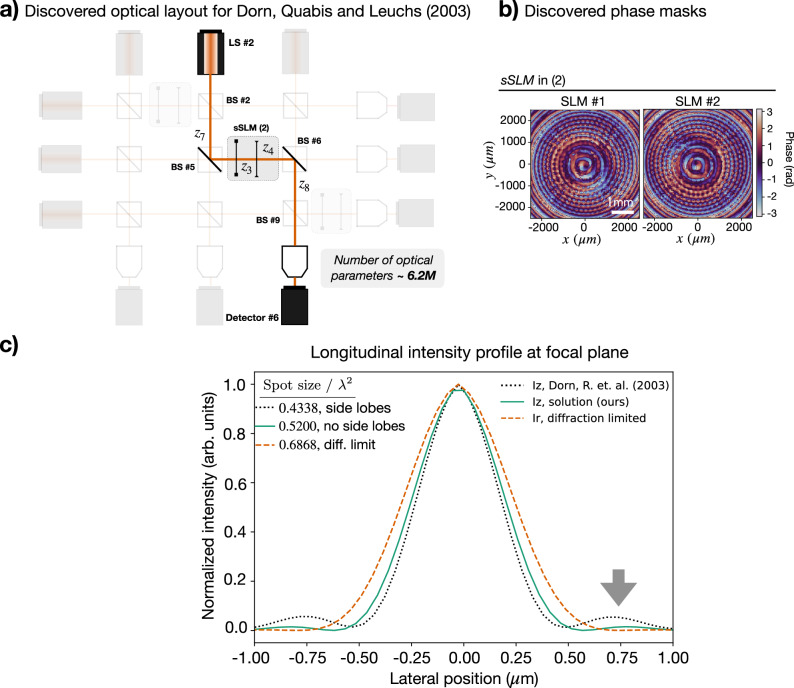
Rediscovery of Dorn, Quabis, and Leuchs (2003)^51^ within highly parameterized optical systems. **a** Discovered virtual optical setup topology. The parameter space  (∼6.2 million optical parameters) is defined by 3 super-SLMs (i.e., 6 SLMs) of (1024 × 1024) pixel resolution and a computational pixel size of 4.8 μm, 9 beam splitter ratios (BS), 8 distances (*z*) and 3 wave plates (with variable phase retardance *η* and orientation angle *θ*). The minimum value of the loss function is demonstrated in detector *#*6. The setup topology is retrieved from detector *#*6 following the identified beam splitter ratios across the system. The identified optical parameters correspond to: the beam splitter ratios, in [Transmittance, Reflectance] pairs: BS*#*2: [0.999, 0.000], BS*#*5: [0.000, 0.999], BS*#*6: [0.000, 0.999], and BS*#*9: [0.999, 0.000]. The wave plate’s *η* = 1.51, *θ* = 3.95; propagation distances (in cm): *z*_3_ = 20.49, *z*_4_ = 63.26, *z*_7_ = 47.92 and *z*_8_ = 31.33. **b** Discovered phase patterns for sSLM *#*2. The speckle-like patterns of SLMs' phase masks are not detrimental to robustness. **c** Normalized longitudinal intensity profile, ∣*E*_*z*_∣^2^, for Dorn, Quabis, and Leuchs (2003) and the identified solution (black dotted, and green lines, respectively) and radial intensity profile, ∣*E*_*x*_∣^2^ + ∣*E*_*y*_∣^2^, of the diffraction-limited linearly polarized beam (orange dotted line). Lateral position indicates lateral distance from the optical axis. The spot size is computed as *ϕ* = (*π*/4)FWHM_*x*_ FWHM_*y*_, where FWHM denotes Full Width Half Maximum. The discovered approach breaks the diffraction limit, demonstrating a larger spot size as the reference. However, it does not feature side lobes (indicated with a gray arrow), which can limit practical imaging techniques.

The discovered topology phase patterns for Dorn, Quabis, and Leuchs are depicted in Fig. 6a and b, respectively. These produce an *LG*_2,1_ Laguerre-Gaussian mode^64^, which demonstrates an intensity pattern of concentric rings with a phase singularity in its center. Surprisingly, XLuminA found an alternative way to imprint a phase singularity onto the beam and produce pronounced longitudinal components on the focal plane. The longitudinal intensity profiles of the discovered solution and the reference experiment are depicted in Fig. 6c.

In both cases XLuminA successfully discovered alternative optical solutions demonstrating similar performance as the reference experiments.

### Discovery of a previously unreported experimental blueprint

**Fig. 7:**
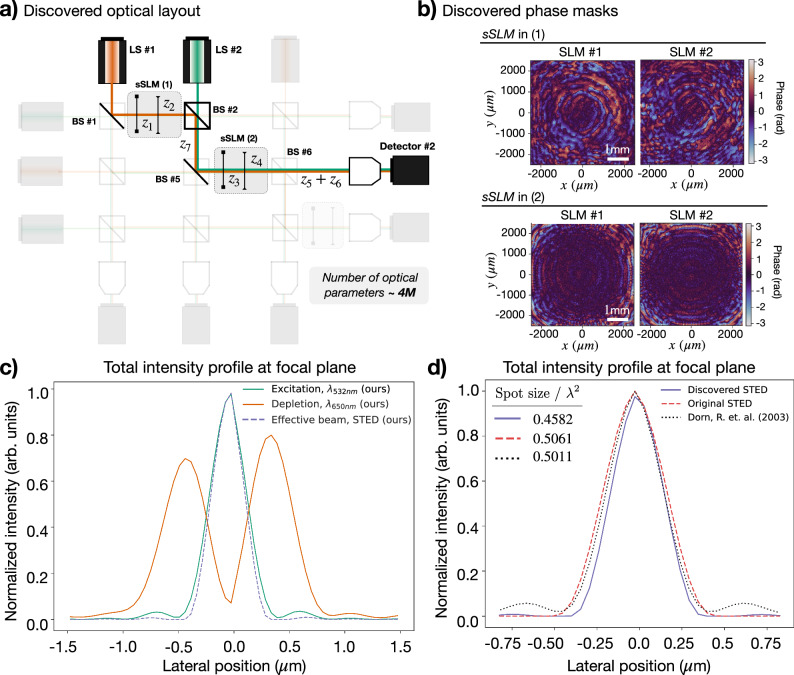
Discovery of a previously unreported experimental blueprint within a highly parameterized optical setup. The parameter space comprises 6 × (824 × 824) pixel phases, 6 extra optical modulation parameters corresponding to the wave plates, and 8 distances (a total of ∼4 million optical parameters). **a** Discovered optical topology. The minimum value of the loss is demonstrated in detector *#*2. The setup topology is easily retrieved from detector *#*2 following the identified beam splitter ratios across the system. The identified optical parameters correspond to: the beam splitter ratios, in [Transmittance, Reflectance] pairs: BS*#*1: [0.000, 0.999], BS*#*2: [0.338, 0.662], BS*#*5: [0.000, 0.999], and BS*#*6: [0.999, 0.000]. The wave plates, in radians (1): *η* = 1.09, *θ* = 0.28, and (2): *η* = 0.19, *θ* = − 3.16. The propagation distances (in cm) are *z*_1_ = 29.98, *z*_2_ = 56.91, *z*_3_ = 58.28, *z*_4_ = 99.91, *z*_5_ = 42.89, *z*_6 _= 53.96, and *z*_7_ = 50.96. **b** Discovered phase masks corresponding to the super-SLM (sSLM) in (1) and (2). The speckle-like patterns of SLMs' phase masks are not detrimental to robustness. **c** Total intensity (∣*E*_*x*_∣^2^ + ∣*E*_*y*_∣^2^ + ∣*E*_*z*_∣^2^) horizontal cross-section of the detected light beams of 650 nm (orange), 532 nm (green), and effective beam emulating stimulated emission (dashed blue). **d** Horizontal cross-section of the normalized total intensity (∣*E*_*x*_∣^2^ + ∣*E*_*y*_∣^2^ + ∣*E*_*z*_∣^2^) of the effective beam from the discovered solution (blue), the simulated STED reference (dashed red), and the simulated reference (dotted black) using 532 nm wavelength. The spot size is computed as *ϕ* = (*π*/4)FWHM_*x*_ FWHM_*y*_, where FWHM denotes Full Width Half Maximum. We use a fluorophore emission wavelength of 560 nm. The discovered solution outperforms both simulated references for STED microscopy^10^ and the sharp focus from Dorn, Quabis, and Leuchs (2003)^51^, demonstrating a spot size of 9.45% smaller than both references.

Finally, we demonstrate the capability of XLuminA for genuine discovery. We initialize the system in the virtual setup depicted in Supplementary Fig. 8. The loss function corresponds to Equation (2), considering the total intensity of the effective beam resulting from the emulated STED process. The details of the optimization are provided in the Supplementary Information section Discovery of a Previously Unreported Experimental Blueprint. The discovered topology and identified phase patterns are depicted in Fig. 7a and b, respectively. The detected intensity topologies reveal the system generates doughnut-shaped and Gaussian-like beams. We compute the vertical cross-section of the focused intensity patterns for both beams and the resulting effective beam (green, orange, and dotted blue lines in Fig. 7c, respectively). The horizontal cross-section exhibits analogous features. We further compare the effective beam intensity with the simulated STED ref. ^10^ and the discovered Gaussian-like beam with the simulated sharp focus ref. ^51^. The obtained results are showcased in Fig. 7d. Strikingly, the discovered solution exploits the underlying physical concepts of two aforementioned optical systems. On the one hand, it generates a doughnut-shaped depletion beam, as demonstrated in ref. ^10^. On the other hand, it generates a Gaussian-like excitation signal with a sharper focus, achieving smaller effective intensity spots resulting from the STED process. The discovered solution showcases an effective beam profile that is sharper than the simulated STED reference. This occurs due to the enhanced sharpening of the longitudinal component of the excitation beam, which demonstrates a similar profile as the simulated sharp focus ref. ^51^. To the best of our knowledge, this technique has never been discussed in the scientific literature before. Human-designed techniques in optics, which we use in this work as baselines, are built on clear concepts. Any minor improvement over well-established baselines indicates unexplored underlying physical techniques from which human scientists can learn new scientific ideas. Regardless of its physical realizability, this solution demonstrates the ability of XLuminA to uncover interesting solutions within highly complex systems.

## Discussion

In this work, we present XLuminA, a highly efficient computational framework with seamlessly integrated auto-differentiation capabilities, just-in-time compilation, automatic vectorization, and GPU compatibility, for the discovery of innovative optical setups in super-resolution microscopy.

We demonstrate the high performance and efficiency of XLuminA with a computational speed-up of × 2.1 ⋅ 10^4^ on GPU, and × 8.4 ⋅ 10^2^ on CPU, compared to standard numerical optimization methods. We further prove the accuracy and reliability of our methods by successfully rediscovering three foundational optical experiments. More significantly, within a large-scale discovery framework, XLuminA identified a previously unreported experimental blueprint that breaks the diffraction limit by integrating the physical principles of two well-known super-resolution techniques.

Having laid the groundwork with XLuminA for an efficient and versatile optics simulator, many other microscopy and imaging techniques follow as a natural extension. In particular, given the modular nature of our framework, additional optical components and physical features can be easily implemented. For example, amplitude modulation, nonlinear interactions, light scattering of probe samples, or time information using algorithms iterating over the simulator. These would enable systems such as iSCAT^65^, structured illumination microscopy^15^, and localization microscopy^66^. Also, inspired by the work in^29^, we could leverage similar approaches to optimize hardware-software discovery within our extensive framework.

In addition, XLuminA provides already the basis for an expansion to complex quantum optics microscopy techniques^67^ or other quantum imaging techniques^68^, as a quantum of light (i.e., a photon) is nothing else than an excitation of the modes of the electromagnetic field. Looking further into the future, one can expect that matter-wave beams (governed by Schrödinger’s equation, which is closely related to the paraxial wave equation, a special case of the electromagnetic field) can be simulated in the same framework. This might allow for the AI-based design of microscopy techniques which could harness entirely new ideas combining light and complex matter wave beams such as electron-beams^69–71^ or coherent beams of high-mass particles^72^. Ultimately, bringing so far unexplored concepts from diverse areas of physics to microscopy applications is at the heart of AI-driven discovery in this area, and we hope that this work constitutes a first step in this direction.

## Methods

### Robustness of the framework

To ensure our methods correctly simulate real-world experimental conditions, we have included noise sources, misalignment, and imperfections in all optical components. The results presented in this work are computed considering uniformly distributed random noise in the phase values for spatial light modulators (SLMs) and wave plates (WP) in the range of ± (0.01 to 0.1) radians, covering all qualities available in current experimental devices. We have incorporated uniformly distributed random misalignment ranging from ± (0.01 to 0.1) millimeters, covering both expert-level precision (± 0.01 mm) and beginner-level accuracy (± 0.1 mm), which is a much larger misalignment than the typically encountered in the lab. Finally, we have included a 1% imperfection on the transmissivity/reflectivity of beam splitters (BS), which is a realistic approach given the high quality of the currently available hardware.

**Fig. 8:**
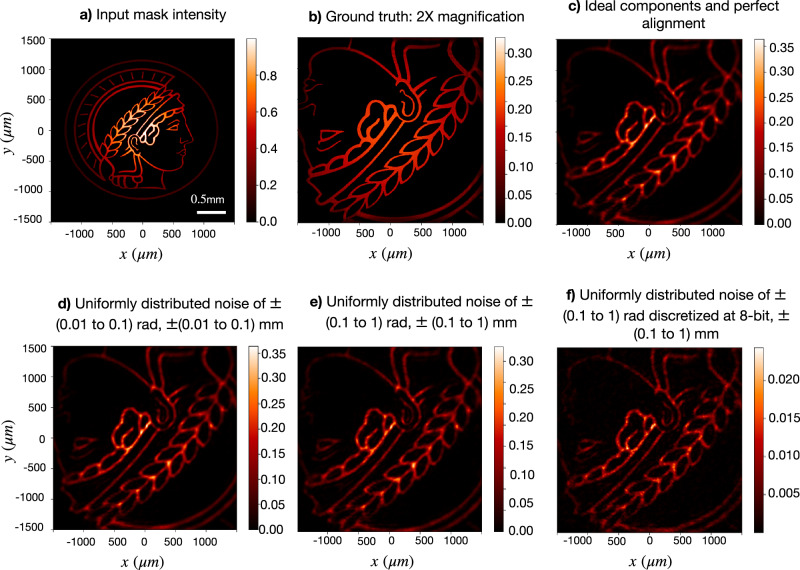
Performance of the discovered optical configuration employed to magnify the image under different levels of noise and misalignment. **a** Input mask intensity. **b** Ground truth intensity pattern (2 × magnification). **c** Detected intensity pattern using the discovered optical system with ideal optical components and perfect alignment. **d** Performance under standard experimental conditions: uniformly distributed random noise of ± (0.01 to 0.1) radians in spatial light modulators (SLMs), and ± (0.01 to 0.1) mm (millimeters) of misalignment. **e** Performance under very large uniformly distributed random noise of ± (0.1 to 1) radians in SLMs and ± (0.1 to 1) mm of misalignment. **f** Performance under very large uniformly distributed random noise of ± (0.1 to 1) radians, discretized at 8-bit, in SLMs and ± (0.1 to 1) mm of misalignment. The discovered system maintains good imaging performance even under very high error conditions, demonstrating its robustness.

We further prove the robustness of our methods by conducting a comparative analysis of the discovered 4f-optical correlator system in Fig. 2 under different noise and misalignment levels using a complex input structure, the Max Planck Society’s logo. The results are depicted in Fig. 8. In particular, Fig. 8a and b showcase the input mask intensity and the desired output image (i.e., computed using the baseline system), respectively. Figure 8c depicts the detected intensity using ideal optical components and no misalignment. Figure 8d shows the detected intensity pattern when including uniformly distributed random noise of ± (0.01 to 0.1) rads in the SLMs and misalignment of ± (0.01 to 0.1) mm. Figure 8e and f depict the detected intensity pattern when including very high, uniformly distributed random noise of ± (0.1 to 1) rads in the SLMs (discretized at 8-bit for Fig. 8d) and misalignment of ± (0.1 to 1) mm.

Clearly, the difference in the detected intensity using ideal optical components and perfect alignment, including standard error sources is negligible (Fig. 8c and d, respectively). Even under very large error conditions (Fig. 8e and f), our framework maintains good imaging performance, which highlights the robustness of our methods.

### Towards large-scale discovery

In this section we first discuss the enormous search space corresponding to large-scale optical setups. Then, we provide the derivation of the loss function in Equation (2). The details about our simulated emission depletion (STED) model, the performance of XLuminA in building our computational ansatz, and running the experiments are provided in the Supplementary Information section Towards Large-Scale Discovery.

#### The large-scale search space

The large-scale optical setup depicted in Fig. 3 consists of 6 light sources that emit linearly polarized Gaussian beams with different wavelengths (e.g., 625 nm, 530 nm, and 470 nm). Through 82 vectorial propagation (vectorial Rayleigh-Sommerfeld, VRS), these beams interact with a total of 9 beam splitters, 24 sSLMs (i.e., 48 SLMs), 24 wave plates, and get ultimately detected by 6 high NA objective lenses focusing on light detectors.

We analyze the number of possible discrete arrangements within this general optical setup. For a very discrete approach of beam splitter ratios (either transmit, reflect, or have light in both arms) and only allowing the SLMs and wave plates (WP) to be switched ON/OFF (i.e., displaying a constant zero phase or adding zero retardance to the incoming light), the number of possible discrete layouts is of

3 $${N}_{{{\rm{Discrete}}}\,{{\rm{layouts}}}}={3}^{{9}_{{{\rm{BS}}}}}\cdot {2}^{4{8}_{{{\rm{SLM}}}}}\cdot {2}^{2{4}_{{{\rm{WP}}}}}=2\cdot 1{0}^{20}.$$

All these are considering that the available beam splitter ratios are restricted to 3 values and the SLMs and wave plates to turn ON/OFF, respectively. In practice, beam splitter ratios and phase values are continuous variables and can take any value (from 0 to 1 and  − *π* to *π*, respectively), which increases even more the dimension of our search space.

In the following Table 1, we present a summary detailing the main properties of the six optimizations conducted within our large-scale ansatz: the number of tunable elements, the dimension of the parameter space and the available number of topologies. As shown in Table 1, our search space typically involves 4 to 6 million optical parameters. In such highly parameterized, non-convex systems, finding the global minimum is generally not feasible. To effectively explore the search space we conduct multiple optimizations with randomly initialized parameters. In addition, we use a momentum-based optimizer (AdamW) to help mitigate convergence issues. While techniques like particle swarm optimization could potentially improve the chances of finding global optimum solutions, our approach leads to good local optima that provide valuable insights into optics.

**Table 1:** Outline of the main properties of the six digital experiments conducted within our large-scale ansatz

Experiment	*#* tunable elements	*#* parameters	*#* topologies
Fig. 4a	21	25	10^5^
Fig. 4c	21	25	10^5^
Fig. 5	26	∼4 million	10^7^
Fig. 6	26	∼6.3 million	10^7^
Fig. 7	26	∼4 million	10^7^
Supplementary Fig. 6	50	52	10^18^

Displays the total number of tunable elements, the dimension of the parameter space, and the available topologies.

#### Loss function

The loss function, ${{\mathcal{L}}}$, is inversely proportional to the total detected intensity density that is above a specified intensity threshold, *I*_*ε*_. Thus, minimizing ${{\mathcal{L}}}$ aims to maximize the generation of small, high-intensity beams. In particular, it reads

4 $${{\mathcal{L}}}=\frac{1}{{{\rm{Density}}}}=\frac{{{\rm{Area}}}}{{I}_{\varepsilon }}.$$

The total intensity *I*_*ε*_ above the threshold is computed as

5 $${I}_{\varepsilon }={\sum }_{k,l}^{N}{i}_{\varepsilon }(k,l),$$

where *N* is the total number of pixels in the camera’s sensor and *i*_*ε*_(*k*, *l*) represents the intensity value at each pixel once the threshold condition is applied. This condition is defined as follows:

6 $${i}_{\varepsilon }(k,l)=\left\{\begin{array}{ll}{i}_{\det }(k,l)\quad &{{\rm{if}}}\,{i}_{\det }(k,l)\, > \, \varepsilon {i}_{\max },\\ 0\hfill\quad &{{\rm{otherwise}}},\end{array}\right.$$

where ${i}_{\det }(k,l)$ is the intensity value at the i-th row and j-th column in the detected 2D intensity pattern, $\varepsilon {i}_{\max }$ (with 0 ≤ *ε* ≤ 1) is the threshold value, with ${i}_{\max }$ being the maximum intensity value in the entire 2D detector array.

The Area is determined using a variation of the Heaviside function Θ applied to *i*_*ε*_, quantifying the area where the intensity is above the threshold:

7 $${{\rm{Area}}}={\sum }_{k,l}^{N}\, \Theta \, ({i}_{\varepsilon }(k,l)),$$

where *N* is the total number of pixels in the camera’s sensor and Θ(*i*_*ε*_(*k*, *l*)) is defined as:

8 $$\Theta ({i}_{\varepsilon }(k,l))=\left\{\begin{array}{ll}1\quad &{{\rm{if}}}\,{i}_{\varepsilon }(k,l)\, > \,0,\\ 0\quad &{{\rm{otherwise}}}.\end{array}\right.$$

Therefore, the loss function can be read as follows:

9 $${{\mathcal{L}}}=\frac{1}{{{\rm{Density}}}}=\frac{{{\rm{Area}}}}{{I}_{\varepsilon }}=\frac{\mathop{\sum }_{k,l}^{N}\Theta ({i}_{\varepsilon }(k,l))}{\mathop{\sum }_{k,l}^{N}{i}_{\varepsilon }(k,l)}.$$

It is important to note that the camera pixel selection in Equation (8) is a discrete operation. However, JAX offers some interesting capabilities due to its integrated autodiff framework. In particular, control flow operations in JAX are supported and differentiable. Therefore, we compute the loss function in a fully differentiable manner using jax.numpy.where thresholding operations maintain differentiability by evaluating both branches (True/False). While this approach is effective, future implementations could benefit from using soft functions such as sigmoids for thresholding, which would provide a smoother transition.

Finally, it is worth mentioning that the derivative of the pixel area (the numerator) is zero. However, the gradient of the loss function is influenced by the numerator, which contributes a global scaling factor (the value of the pixel area), which value changes over-optimization iterations. This design is important as it dynamically adjusts the gradients (it contributes to having larger gradients for larger pixel areas and smaller gradients for smaller areas) and effectively guides the optimization toward maximizing intensity in small pixel regions.

Crucially, light is detected across six different devices. Therefore, we compute the loss function at each detector and the parameter update is driven by the detector that shows the minimum value of the loss. We conduct this selection by using a differentiable, smooth approximation using jax.nn.logsumexp() as:

def softmin(l_det, beta): return - logsumexp(-beta * l_det)/ beta,

where l_det is the array of the loss values corresponding to each detector, and beta is the strength of the modulation.

## Supplementary information

Supplementary Information

Transparent Peer Review file

## Data Availability

Data can be readily generated using the Python scripts provided via GitHub at https://github.com/artificial-scientist-lab/XLuminA.

## Code availability

The produced code and documentation can be found via GitHub at https://github.com/artificial-scientist-lab/XLuminA.
