# Supplementary material for: Automated discovery of experimental designs in super-resolution microscopy with XLuminA
Source: Nat Commun. 2024 Dec 10;15:10658. doi: 10.1038/s41467-024-54696-y (PMC11632100; doi:10.1038/s41467-024-54696-y)
Supplement: Supplementary file 1 — Supplementary Information [file 41467_2024_54696_MOESM1_ESM.pdf]

Automated Discovery of Experimental Designs in Super-Resolution Microscopy with XLuminA

Carla Rodríguez,[1,*] Sören Arlt,[1] Leonhard Möckl,[1,2,3,4] and Mario Krenn[1]

[1] *Max Planck Institute for the Science of Light, Erlangen, Germany*
[2] *Friedrich-Alexander-University Erlangen-Nuremberg, Faculty of Sciences, Department of Physics, Erlangen, Germany*
[3] *Friedrich-Alexander-University Erlangen-Nuremberg, Faculty of Medicine 1/CITABLE, Erlangen, Germany*
[4] *Deutsches Zentrum Immuntherapie (DZI), Erlangen, Germany*

I. FEATURES AND PERFORMANCE OF XLUMINA

In this section we provide the detailed description of XLuminA's simulation features and performance. All the experiments were run on an Intel CPU Xeon Gold 6130 and Nvidia GPU Quadro RTX 6000. The simulator enables, among many other features, to define light sources (of any wavelength and power), phase masks (i.e., spatial light modulators, SLMs), polarizers, variable retarders (e.g., wave plates, WPs), diffraction gratings, and high numerical aperture (NA) lenses to replicate strong focusing conditions. Light propagation and diffraction is simulated by two methods, each available for both scalar and vectorial regimes: the fast-Fourier-transform (FFT) based numerical integration of the Rayleigh-Sommerfeld (RS) diffraction integral [1, 2] and the Chirped z-transform (CZT) [3]. The CZT is an accelerated version of the RS algorithm, which allows for arbitrary selection and sampling of the region of interest. These algorithms are based on the FFT and require a reasonable sampling for the calculation to be accurate [4]. In our simulations we consider light sources emitting Gaussian beams of 1.2 mm beam waist. To avoid possible boundary-generated artifacts during the simulation, we define these beams in larger computational spaces of 4 mm or 5 mm. Thus, the pixel resolutions often span 1024×1024, or 2048×2048.

Some functionalities of XLuminA's optics simulator (e.g., optical propagation algorithms, planar lens or amplitude masks) are inspired in an open-source NumPy-based Python module for diffraction and interferometry simulation, Diffractio [5], although we have rewritten and modified these approaches to combine them with JAX just-in-time (jit) functionality. In essence, jit compilation optimizes sequences of operations together and runs them at once. For this purpose, the first run of a jitted function builds an abstract representation of the sequence of operations specified by the function. This representation encodes the shape and the data-type (dtype) of the arrays - but is agnostic to the values of such arrays. If the input shapes and dtypes are not modified, the abstract structure of the function can be then re-used for subsequent runs, without re-compilation, which allows to execute the subsequent calls faster. However, if the input shape or dtype is modified, the function automatically gets re-compiled. This will cause an extra overhead time due to the extraction of a new abstract structure of the function for the new shapes/dtypes. On top of that, we developed specialized functions (e.g., beam splitters, WPs or propagation through high NA objective lens with CZT methods, to name a few) which significantly expand the software capabilities. The most important hardware addition on the optical simulator are the SLMs, each pixel of which possesses an independent (and variable) phase value. They serve as a universal approximation for phase masks, including lenses, and offer a computational advantage: given a specific pixel resolution, they allow for unrestricted phase design selection. Such flexibility is crucial during the parameter space exploration, as it allows the software to autonomously probe all potential solutions. In addition, we defined under the name of super-SLM (sSLM) a hardware-box-type which consists of two SLMs, each one independently imprinting a phase mask on the horizontal and vertical polarization components of the field each.

We first evaluate the performance of our optimized functions against their counterparts in Diffractio [5]. The acquired average execution time over 100 runs, within a resolution of 2048×2048 pixels, for scalar and vectorial field propagation using Rayleigh-Sommerfeld (RS, VRS) and Chirped z-transform (CZT, VCZT) algorithms, are presented in Fig. 1b of the main manuscript. The single run data is depicted in Supplementary Figs. 1 and 2.

* carla.rodriguez@mpl.mpg.de

To evaluate the performance of numerical and auto-differentiation methods we chose to use Broyden-Fletcher-Goldfarb-Shanno (BFGS) algorithm [6] (from SciPy's Python library [7]) and Adam [8] (included in the JAX library) as optimizers. As the optical system, we set-up a Gaussian beam propagating over a distance z and interacting with a phase mask. The objective function is the mean squared error between the detected light and the ground truth, characterized by a Gaussian beam with a spiral phase imprinted on its wavefront. We initialize the system with an arbitrary phase mask configuration.

First, we evaluate the computational time for a single gradient evaluation for numerical and autodiff methods across different computational window sizes (from 10×10 up to 500×500 pixels) and devices (CPU and GPU). We keep the default settings for BFGS. For Adam, the step size is set to 0.1. The optimization process is terminated if there is no improvement in the loss value (meaning it has not decreased below the best value recorded), over 50 consecutive iteration steps. For each resolution window, we collect the convergence time of both optimizers and divide it by the total number of gradient evaluations for BFGS and the total number of steps for Adam. The acquired gradient evaluation times correspond to the mean value over 5 runs. The speed-up magnitude for different pixel resolutions is depicted in Supplementary Fig. 3a. Clearly, autodiff outperforms numerical methods by up to 4 orders of magnitude on CPU and 5 orders of magnitude when running in the GPU. The advantage over larger sizes is clear given that we run simulations with resolutions of 1024×1024 and 2024×2048 pixels.

Finally, we conduct the evaluation of the convergence time for both methods. We keep the aforementioned settings for the optimizers. We initialize the systems 5 times and compute their mean value. The acquired results are depicted in Fig. 1d of the main manuscript. On the CPU, numerical methods exhibit exponential scaling in convergence time, reaching about $4.5 \cdot 10^4$ seconds (roughly 12 hours) for 250×250 pixel resolution. In contrast, autodiff demonstrates superior efficiency, reducing it to roughly 53 seconds. GPU optimization performance is even more pronounced, reaching convergence in 0.24 seconds for 250×250 pixels, and 16 seconds for a resolution of 500×500. The speed-up magnitude for different pixel resolutions is depicted in Supplementary Fig. 3b.

For a more general comparison, the performance of XLuminA (autodiff) compared to Diffractio (numerical methods) across different resolutions and optimizers is presented in the Supplementary Fig. 4. Numerical differentiation is computed using Diffractio's optical simulator and the Broyden-Fletcher-Goldfarb-Shanno (BFGS) optimizer and auto-differentiation on XLuminA. We include evaluations using Stochastic-Gradient-Descent (SGD), Adaptive Gradient (AdaGrad), Adaptive moment estimation with weight decay (AdamW) and Adaptive moment estimation (Adam). The step size is set to 0.1 and is common to all the optimizers. For AdamW, the weight decay is set to 10^{-4}. The stopping condition is common to all the frameworks: the optimization is terminated if there is no improvement in the loss value (i.e., it has not decreased below the best value recorded), over 500 consecutive iteration steps. This condition is checked every 100 steps.

From Supplementary Fig. 4 is clear that the use of XLuminA with autodiff methods improves the gradient evaluation time by a factor of $\times 2.4 \cdot 10^5$ in the GPU and a factor of $\times 1.2 \cdot 10^4$ on the CPU for resolutions of 150×150 pixels. This behavior is common to all the tested optimizers (Adam, AdamW, SGD and AdaGrad). When evaluating the convergence time, the use of Autodiff methods on XLuminA using the Adam and AdamW optimizers improve the performance with respect to numerical methods by a factor of $\times 1.1 \cdot 10^4$ and $\times 6.5 \cdot 10^2$ in the GPU, respectively, for a resolution of 150×150 pixels. The performance of Adam and AdamW in the CPU demonstrates factors of $\times 5.8 \cdot 10^2$ and $\times 2.9 \cdot 10^1$, respectively, for the same resolution. The use of Diffractio with numerical methods (BFGS) outperforms both AdaGrad and SGD in convergence time. In particular, numerical methods outperform AdaGrad by a factor of $\times 1.53$ in the GPU and $\times 14.14$ in the CPU, for a resolution of 150×150 pixels. Similar behavior is demonstrated for SGD: numerical methods outperform it by a factor of $\times 1.50$ in the GPU and $\times 43.50$ in the CPU, for a resolution of 150×150 pixels. Overall, the use of Autodiff methods (in particular, using Adam or AdamW) within GPU-accelerated frameworks is a more appropriate choice to conduct efficient optimization.

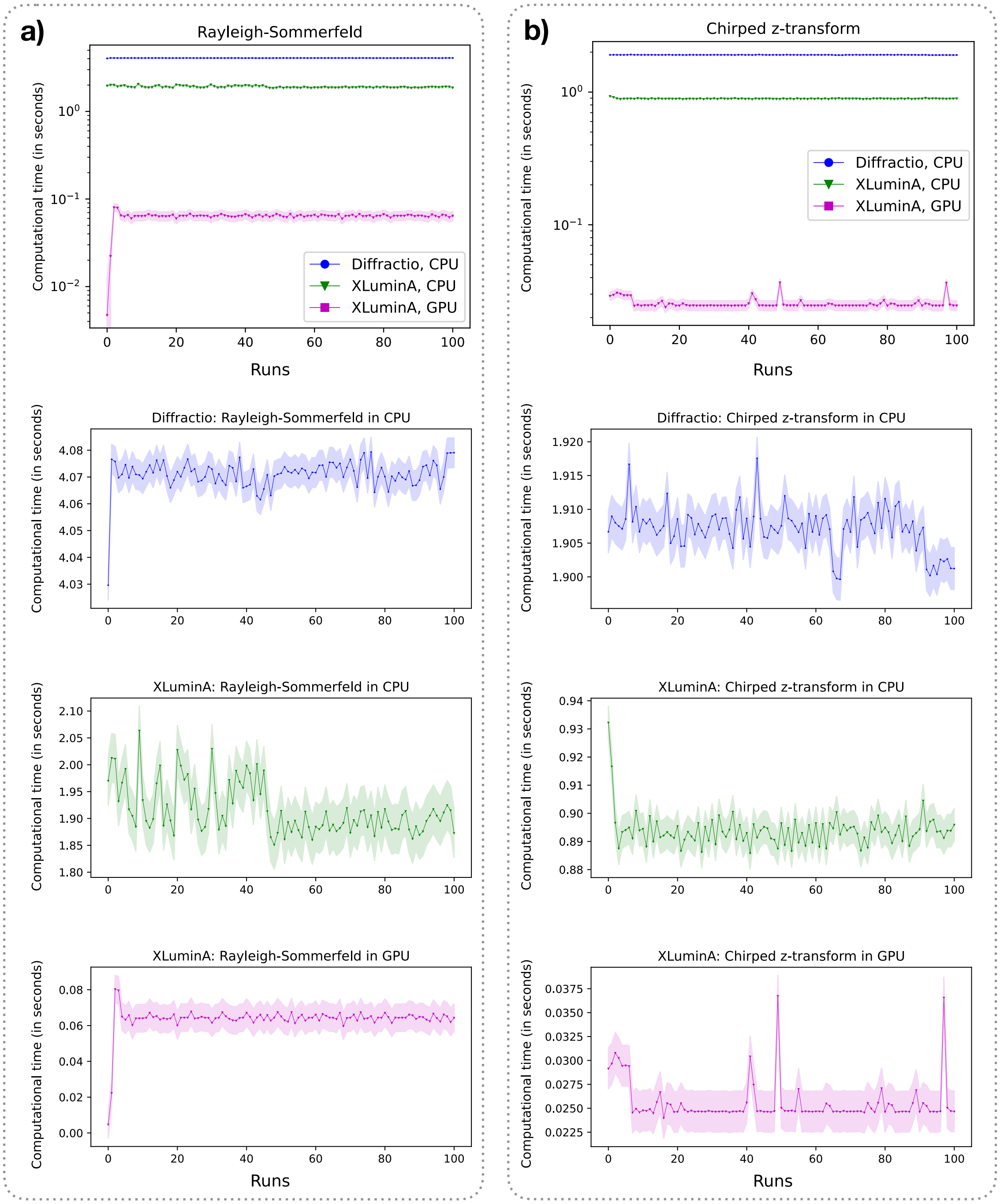

Supplementary Fig. 1. Execution time over 100 runs, within a resolution of 2048 × 2048 pixels for the propagation methods of (a) Rayleigh-Sommerfeld and (b) Chirped z-transform. Times for Diffractio are depicted in blue dots. First row corresponds to the log-scale representation. Times for XLuminA on CPU and GPU correspond to green triangles and magenta squares, respectively. Standard deviation values correspond to shaded regions. Among the minor oscillations of the running times, the Rayleigh-Sommerfeld algorithm behavior stands out. When running in Diffractio, it shows an increase of the execution time of 0.005 seconds from the first run to subsequent runs. Similar behavior can be observed for the Rayleigh-Sommerfeld (RS) algorithm in XLuminA when executing in the GPU. This time, however, the increase occurs during the first 5 runs, from an first execution time of 0.0047 seconds to stabilize in 0.06 seconds for subsequent runs. This behavior is not present when executing XLuminA's RS in the CPU. The origin of this factor of 10 is still unknown. We believe a further optimization on the RS propagation algorithm will improve the execution time.

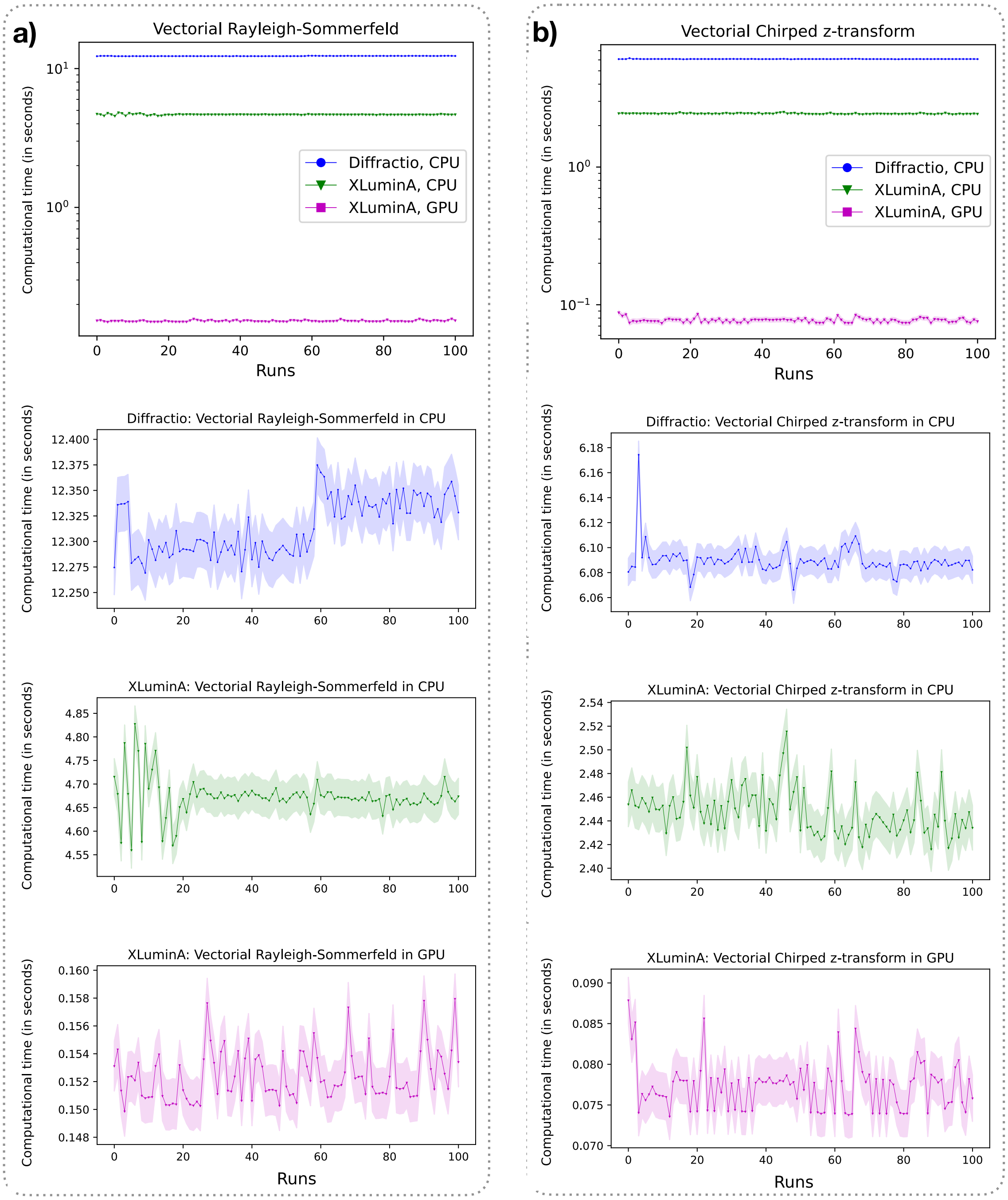

Supplementary Fig. 2. Execution time over 100 runs, within a resolution of 2048×2048 pixels for the propagation methods of (a) Vectorial Rayleigh-Sommerfeld and (b) Vectorial Chirped z-transform. Times for Diffractio are depicted in blue dots. First row corresponds to the log-scale representation. Times for XLuminA on CPU and GPU correspond to green triangles and magenta squares, respectively. Standard deviation values correspond to shaded regions.

II. DATA-DRIVEN REDISCOVERY

In this section we provide the details of the data-driven learning approach outlined in Fig. 2b of the main manuscript. The training dataset is composed of 18,000 [input, output] intensity sample pairs. Each sample consists of a Gaussian beam shaped by amplitude masks in various forms (circles, rectangles, squares and rings), with varying sizes and orientations. The corresponding output for each input is an inverted version, magnified by a factor of 2, using homogeneous intensity distribution.

The training process involves feeding the input mask into the virtual optical setup. The cost function for each optical setup is computed as the mean squared error between the detected intensity pattern from the virtual setup and the corresponding target mask from the dataset. We select training examples in batches of 10 and evaluate the current setup response and its loss value. The average loss over the batch guides the update of the optical parameters, repeating this cycle until convergence is reached. The parameter space (of $\sim$ 2 million optical parameters) includes the three distances and the phase masks of the two SLMs with a resolution of 1024×1024 pixels.

We set-up the AdamW optimizer with a step size of 0.01 and a weight decay of 10^{-4}. The optimization is terminated if there is no improvement in the loss value (i.e., it has not decreased below the best value recorded), over 500 consecutive iteration steps. This condition is checked every 500 steps. We start the optimization with randomly initialized optical parameters with values between 0 and 1. The discovered solution was identified in about 2.4 hours on the GPU. The loss value evolution over the number of iteration steps is depicted in Supplementary Fig. 9a.

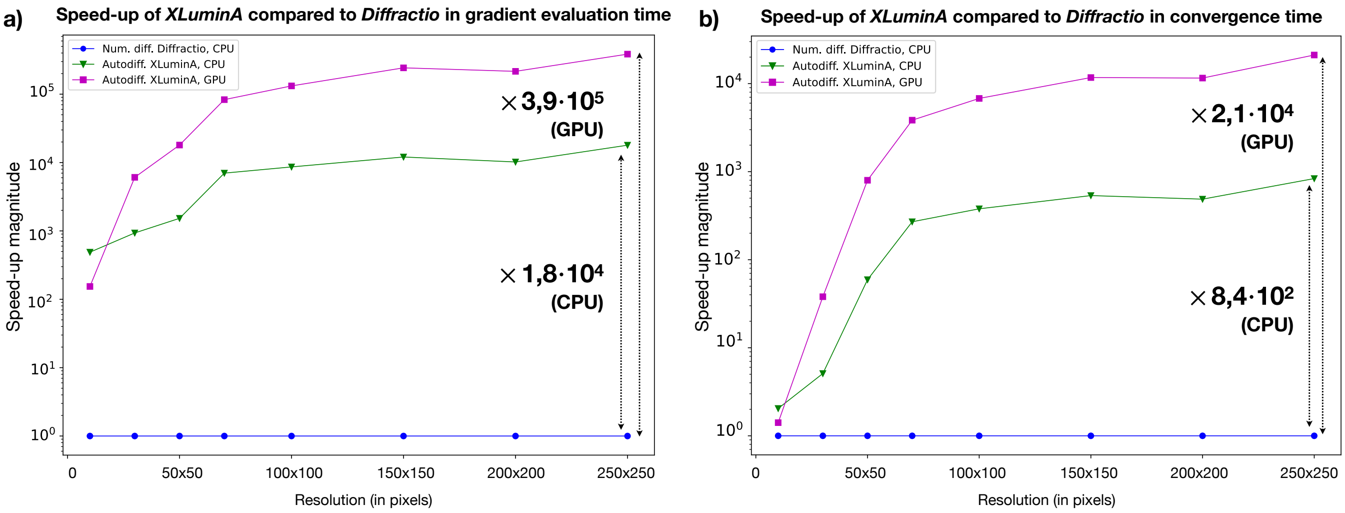

Supplementary Fig. 3. Speed-up magnitudes of XLuminA (auto-differentiation) compared to Diffractio (numerical methods) across different resolutions in (a) single gradient evaluation and (b) convergence time. Numerical differentiation methods are computed using Diffractio's optical simulator (blue dots) and auto-differentiation (green triangles for CPU and magenta squares for GPU) on XLuminA. Autodiff. outperforms numerical methods in gradient evaluation by up to 4 orders of magnitude when running on CPU and 5 orders of magnitude when running on the GPU for a resolution of 250×250 pixels. In convergence time, autodiff outperforms numerical methods by almost 3 orders of magnitude in the CPU and 4 orders of magnitude on the GPU.

III. TOWARDS LARGE-SCALE DISCOVERY

In this section we first evaluate the performance of XLuminA in building our large-scale computational ansatz. Then, we detail the methodology for the optimizations conducted using our quasi-universal computational ansatz, a purely continuous framework.

In particular, we provide the details for running the optimizations for STED microscopy and the super-resolution technique exploiting light vortices in two different scenarios, using: (1) optimizable topologies and optical parameters within low parameterized systems, and (2) optimizable topologies and optical parameters within highly parameterized, complex optical setups.

A. Performance on large-scale optical setups

We evaluate the efficiency of XLuminA by measuring the computational time it takes to construct the large-scale optical setup depicted in Fig. 3 of the main manuscript and comparing it with respect to Diffractio's simulation across different resolutions (from 10×10 to 1024×1024 pixels) and devices (CPU and GPU). The resulting times, measured across varying computational window sizes, are depicted in Supplementary Fig. 5.

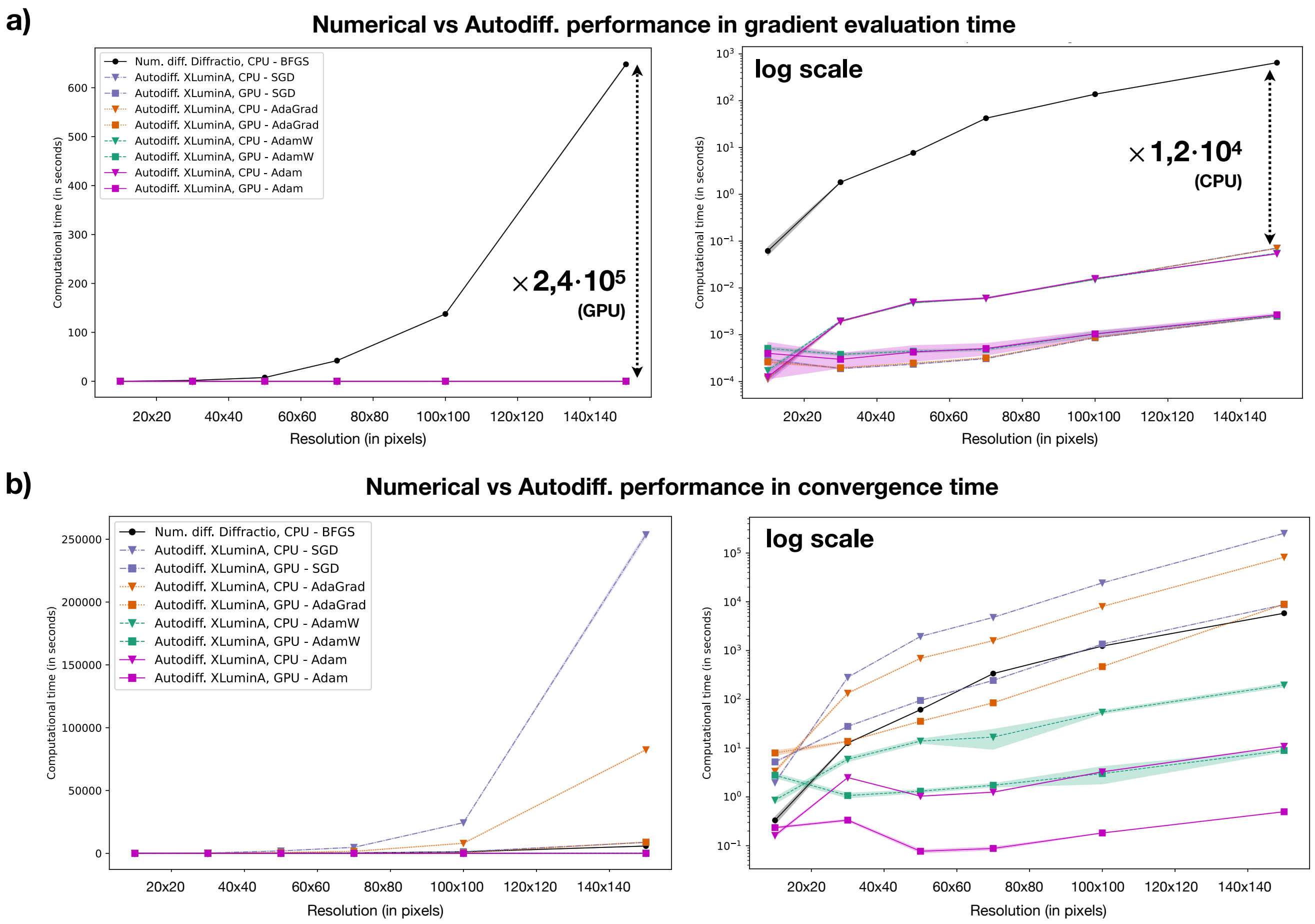

Supplementary Fig. 4. Performance of XLuminA (auto-differentiation) compared to Diffractio (numerical methods) across different resolutions and optimizers in (a) single gradient evaluation and (b) convergence time. Data corresponds to the average time over 5 runs. Numerical differentiation is computed using Diffractio's optical simulator and the Broyden-Fletcher-Goldfarb-Shanno (BFGS) optimizer (black dots) and auto-differentiation (triangles for CPU and squares for GPU) on XLuminA. The Stochastic-Gradient-Descent (SGD), Adaptive Gradient (AdaGrad), Adaptive moment estimation with weight decay (AdamW) and Adaptive moment estimation (Adam) correspond to blue (dash-dot line), orange (dotted line), green (dash line) and magenta (continuous line), respectively. Shaded regions correspond to standard deviation values. The use of XLuminA with autodiff methods improves the gradient evaluation time by a factor of $\times 2.4 \cdot 10^5$ in the GPU and a factor of $\times 1.2 \cdot 10^4$ on the CPU for resolutions of 150×150 pixels. This behavior is common to all the tested optimizers (Adam, AdamW, SGD and AdaGrad). When evaluating the convergence time, the use of Autodiff methods on XLuminA using the Adam and AdamW optimizers improve the performance with respect to numerical methods by a factor of $\times 1.1 \cdot 10^4$ and $\times 6.5 \cdot 10^2$ in the GPU, respectively, for a resolution of 150×150 pixels. The performance of Adam and AdamW in the CPU demonstrates factors of $\times 5.8 \cdot 10^2$ and $\times 2.9 \cdot 10^1$, respectively, for the same resolution. Overall, the use of Autodiff methods (in particular, using Adam or AdamW) within GPU-accelerated frameworks is a more appropriate choice to conduct efficient optimization.

Diffractio exhibits a notable exponential increase in its computational time past the size of 300×300 pixels, showing running times of almost 6 minutes for a resolution of 1024×1024 pixels. For smaller sizes, the efficiency of NumPy's dispatch overhead times becomes relevant, making it faster in such regimes. In fact, when considering resolutions up to 200×200 pixels, XLuminA experiences significant overhead times (on both CPU and GPU). These are attributed to JAX's dispatch overhead. Although this time is independent of the size of the arrays, it becomes more pronounced when multiple operations are performed on smaller arrays. However, as array size increases, the dispatch costs diminish, highlighting the benefits of JAX's accelerated linear algebra and just-in-time compilation.

When running on CPU, XLuminA outperforms Diffractio, exhibiting superior scalability in both initial and subsequent runs. For example, for 1024×1024 pixels, XLuminA operates in nearly half the time (2.5 minutes) required by Diffractio. This advantage becomes even more pronounced when XLuminA operates on a GPU. The initial run times remain fairly consistent across various computational window sizes, ranging from 14 to 16 seconds. Subsequent runs exhibit similar consistency, with times around 3 to 3.7 seconds up to 500×500 pixels and 6 seconds for 1024×1024 pixels.

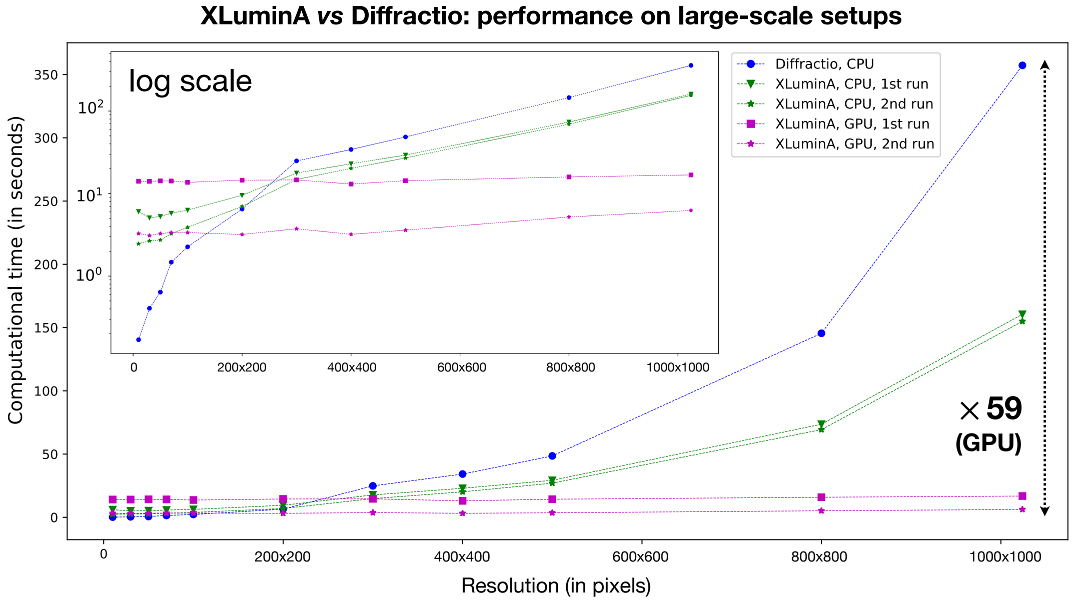

Supplementary Fig. 5. Computational time (in seconds) for generating the large-scale setup for Diffractio (blue dots) and XLuminA across different resolutions on CPU (green triangles) and GPU (magenta squares). For XLuminA, first run curves are decorated with triangles and second runs (pre-compiled jitted functions) with stars. The first run of a jitted function encodes, in an abstract representation, the shape of the arrays (i.e., pixel resolution). If the input shape remains constant, such abstract structure is re-used and there is no need of re-compiling, which allows to execute the subsequent runs faster. Diffractio exhibits a notable exponential increase in its computational time, showing running times of almost 6 minutes for a resolution of 1024×1024. When running on CPU, XLuminA outperforms Diffractio, exhibiting superior scalability in both initial and subsequent runs. For example, for 1024×1024 pixels, XLuminA operates in nearly half the time (2.5 minutes) required by Diffractio. This advantage becomes even more pronounced when XLuminA operates on the GPU, demonstrating a speedup factor of $\times 59$ for a resolution of 1024×1024 pixels.

B. Stimulated emission depletion model

STED microscopy [9, 10] is based on excitation and spatially targeted depletion of fluorophores. In order to achieve this, a Gaussian-shaped excitation beam and a doughnut-shaped depletion beam (generated by imprinting a spiral phase into its wavefront) are concentrically overlapped. The depletion beam has zero intensity in the center, where the excitation beam has its maximum. Fluorophores that are not in the center of the beams are forced to emit at the wavelength of the depletion beam. Their emission is spectrally filtered out. Only fluorophores in the center of the beams are allowed to fluoresce normally, and only their emission is ultimately detected. This effectively reduces the area of normal fluorescence, which leads to super-resolution imaging.

We simulate one of the fundamental concepts of STED microscopy without having to rely on time-dependent

processes related to absorption and fluorescence. To do so, we perform a nonlinear modulation of the intensity of the excitation and depletion beams based on the Beer-Lambert law [11]. We define the effective fluorescence that would ultimately be detected as:

$$I_{\text{eff}} = I_{\text{ex}} \left[1 - \beta \left(1 - e^{-(I_{\text{dep}}/I_{\text{ex}})} \right) \right] \ , \tag{1}$$

where I_{ex} and I_{dep} correspond to the excitation and depletion intensities, respectively, and $0 \leq \beta \leq 1$ captures the quenching efficiency of the depletion beam. This expression bounds the effect of the depletion beam such that scenarios with negative effective intensity or unrealistically high values are avoided. In particular, assuming a perfect efficiency of the depletion beam in suppressing the excitation (i.e., $\beta = 1$), we obtain an expression resembling the Beer-Lambert law:

$$I_{\text{eff}} = I_{\text{ex}} \cdot e^{-(I_{\text{dep}}/I_{\text{ex}})} \ . \tag{2}$$

Thus, the effective detected light falls off exponentially with the intensity ratio $I_{\text{dep}}/I_{\text{ex}}$. In the limit case where there is no excitation intensity, $I_{\text{ex}} = 0$, the detected light is zero as well, $I_{\text{eff}} = 0$. If there is no depletion intensity, $I_{\text{dep}} = 0$, the detected light corresponds to the excitation beam $I_{\text{eff}} = I_{\text{ex}}$. The trivial case of null efficiency in the quenching, $\beta = 0$, leads to the same result.

To evaluate the nonlinear effect we consider $\beta = 1$ and $I_{\text{dep}} = \frac{1}{2} I_{\text{ex}}$. From Equation (1) we obtain

$$I_{\text{eff}} = I_{\text{ex}} e^{-1/2} \approx 0.6 I_{\text{ex}} \ . \tag{3}$$

Now, by slightly increasing the depletion energy, e.g., $I_{\text{dep}} = \frac{3}{2} I_{\text{ex}}$, it reads

$$I_{\text{eff}} = I_{\text{ex}} e^{-3/2} \approx 0.2 I_{\text{ex}} \ . \tag{4}$$

Therefore, a small change in the depletion energy causes a large effect in the effective intensity. As a further example, if we set an intermediate efficiency of $\beta = 0.5$ and $I_{\text{dep}} = \frac{1}{2} I_{\text{ex}}$ we obtain

$$I_{\text{eff}} = I_{\text{ex}} \left[\frac{1 + e^{-1/2}}{2} \right] \approx 0.8 I_{\text{ex}} \ . \tag{5}$$

which clearly demonstrates the effect of diminishing the efficiency of the suppression. Overall, we successfully imprinted the nonlinear behavior of the quenching for different range of effectiveness, achieving a realistic, bounded physical model for STED.

C. Rediscovery through exploration of optical topologies

To further prove the capability of XLuminA to discover experimental setup optical topologies of already existing solutions, we conduct two optimizations using our quasi-universal optical setup scheme. The goal is to discover the optical topology using the available optimizable optical parameters (i.e., distances, beam splitter ratios and wave plate's angles).

Crucially, light is detected across six different devices. Therefore, we compute the loss function at each detector and the parameter update is driven by the detector that shows the minimum value of the loss. We conduct this selection by using a differentiable, smooth approximation using `jax.nn.logsumexp()` as:

```
def softmin(l_det, beta):
    return - logsumexp(-beta * l_det)/ beta,
```

where `l_det` is the array of the loss values corresponding to each detector and `beta` is the strength of the modulation.

We first target XLuminA to discover experimental setup topologies for STED microscopy [9] and Dorn, Quabis and Leuchs (2003) [12] using a virtual optical table featuring randomly positioned phase masks displaying fixed (non-optimizable) phase patterns.

In particular, to rediscover the concept of STED microscopy, we initialize the 3×3 optical setup in Supplementary Fig. 6a, using light sources emitting linearly polarized light of 632.8 nm and 530 nm wavelengths and four phase

masks of 824×824 pixel resolution (PM#1 to PM#4 in Supplementary Fig. 6a) displaying the fixed phase patterns depicted in Supplementary Fig. 6c.

The parameter space (of 25 optical parameters) is defined by 9 symmetric beam splitter ratios, 8 distances and 4 wave plates with variable retardance and orientation (BS#1 to BS#9, z_1 to z_8 and WP#1 to WP#4 in Supplementary Fig. 6a, respectively). The loss function corresponds to

$$\mathcal{L} = \frac{1}{\text{Density}} = \frac{\text{Area}}{I_\varepsilon} \tag{6}$$

where I_ε is the sum of pixel intensity values greater than the threshold value $\varepsilon \cdot i_{\max}$, where $0 \leq \varepsilon \leq 1$ and i_{max} corresponds to the maximum detected intensity. The Area corresponds to the total number of camera pixels fulfilling the same condition. The intensity corresponds to the radial component of the effective beam resulting from the STED process, $|E_x|^2 + |E_y|^2$ and $\epsilon = 0.5$. We simulate the stimulated emission depletion effect using Supplementary Equation (1) with $\beta = 1$. We set-up the AdamW optimizer with a step size of 10^{-3} and a weight decay of 10^{-4}. The system is initialized with random optical parameters with values between 0 and 1. The optimization is terminated if there is no improvement in the loss value over 500 consecutive iteration steps. This condition is checked every 100 steps. The loss value evolution over the number of iteration steps is depicted in Supplementary Fig. 9b. The system converged (in roughly 35 minutes using a GPU) into the topology highlighted in Fig. 4a of the main manuscript, demonstrating the smallest loss value in the detector #3. The identified optical parameters correspond to: the beam splitter ratios, in [Transmittance, Reflectance] pairs: BS#2: [0.999, 0.000], BS#4: [0.926, 0.073], BS#5: [0.998, 0.001], BS#6: [0.001, 0.998], BS#8: [0.342, 0.658], and BS#9: [0.664, 0.336]. The wave plate, in radians (2): $\eta = 1.911$, $\theta = -1.697$. The propagation distances (in cm) are $z_1 = 74.70$, $z_2 = 34.31$, $z_3 = 59.07$, $z_4 = 27.13$, $z_5 = 36.13$, $z_6 = 18.69$, $z_7 = 76.03$ and $z_8 = 20.88$. To retrieve the discovered topology, one can identify the optical path following the light, in reverse, from the detector #3. First, the beam splitter $BS\#9$ has 66.4% transmittance, which implies that light is coming from $BS\#8$ and $BS\#6$. In one hand, $BS\#8$ has a reflectance value of 65.8%, meaning that most of the light comes from $BS\#5$. On the other hand, $BS\#6$ has a reflectance value of 99.8%, meaning that light also comes from $BS\#5$ which, in turn, has a transmittance value of 99.8%. Due to $BS\#5$ being fully transmissible, light paths can be easily redirected to $BS\#2$ and $BS\#4$, with transmission values of 99.9% and 92.6%, respectively. Thus, the Gaussian beam emerging from source #2 goes through the setup without being modulated and light from source #5 interacts with phase mask #2, generating a doughnut-shaped beam in the detector #3.

To rediscover the physical principle exploited in Dorn, Quabis and Leuchs (2003) we initialize the 3×3 optical setup in Supplementary Fig. 6b, using light sources emitting linearly polarized light of 632.8 nm wavelength and four phase masks (of 1024×1024 pixel resolution) displaying the fixed phase patterns depicted in Supplementary Fig. 6b. The parameter space (25 optical parameters) is defined by 9 symmetric beam splitter ratios, 8 distances and 4 wave plates (retardance and orientation). The loss function corresponds to Supplementary Equation (6) considering the intensity of the electromagnetic field's longitudinal component, $|E_z|^2$, and $\epsilon = 0.7$. We set-up the AdamW optimizer with a step size of 10^{-2} and a weight decay of 10^{-4}. The system is initialized with random optical parameters with values between 0 and 1. The optimization is terminated if there is no improvement in the loss value over 500 consecutive iteration steps. This condition is checked every 100 steps. The loss value evolution over the number of iteration steps is depicted in Supplementary Fig. 9c. The system converged (in roughly 1 hour using a GPU) into the topology highlighted in Fig. 4c of the main manuscript, demonstrating the smallest loss value in the detector #3. The identified optical parameters correspond to: the beam splitter ratios, in [Transmittance, Reflectance] pairs: BS#1: [0.000, 0.999], BS#2: [0.000, 0.999], BS#4: [0.037, 0.963], BS#5: [0.999, 0.000], BS#6: [0.030, 0.970], BS#8: [0.001, 0.999], and BS#9: [0.589, 0.411]. The wave plates, in radians (1): $\eta = -0.027$, $\theta = -1.991$; and (2): $\eta = -4.698$, $\theta = -1.551$. The propagation distances (in cm) are $z_1 = 15.27$, $z_2 = 46.46$, $z_3 = 16.08$, $z_4 = 90.66$, $z_5 = 173.84$, $z_6 = 121.16$, $z_7 = 30.07$ and $z_8 = 70.09$. Alike for the previous example, we retrieve the discovered topology following the light from the detector #3. First, the beam splitter $BS\#9$ has 58.9% transmittance, which implies that light is coming from $BS\#8$ and $BS\#6$. In one hand, $BS\#8$ has a reflectance value of 99.9%, meaning that light comes from $BS\#5$. On the other hand, $BS\#6$ has a reflectance value of 97.0%, meaning that light also comes from $BS\#5$ which, in turn, has a transmittance value of 99.9%. Due to $BS\#5$ being fully transmissible, light paths can be easily redirected to $BS\#2$ and $BS\#4$, with reflectance values of 99.9% and 96.3%, respectively. Which leads to $BS\#1$ with a transmittance value of 99.9%. Thus, the light emerging from sources #1 and #4 interacts, independently, with the phase masks #1 and #2, respectively, imprinting phase singularities in the center of the beam generating doughnut-shaped beams. Light gets ultimately combined before detector #6.

Importantly, we are not restricted to the use of 3×3 optical grids. Thus, we conduct the same optimization procedure for Dorn, Quabis and Leuchs (2003) this time within a 6×6 optical system. The obtained results are

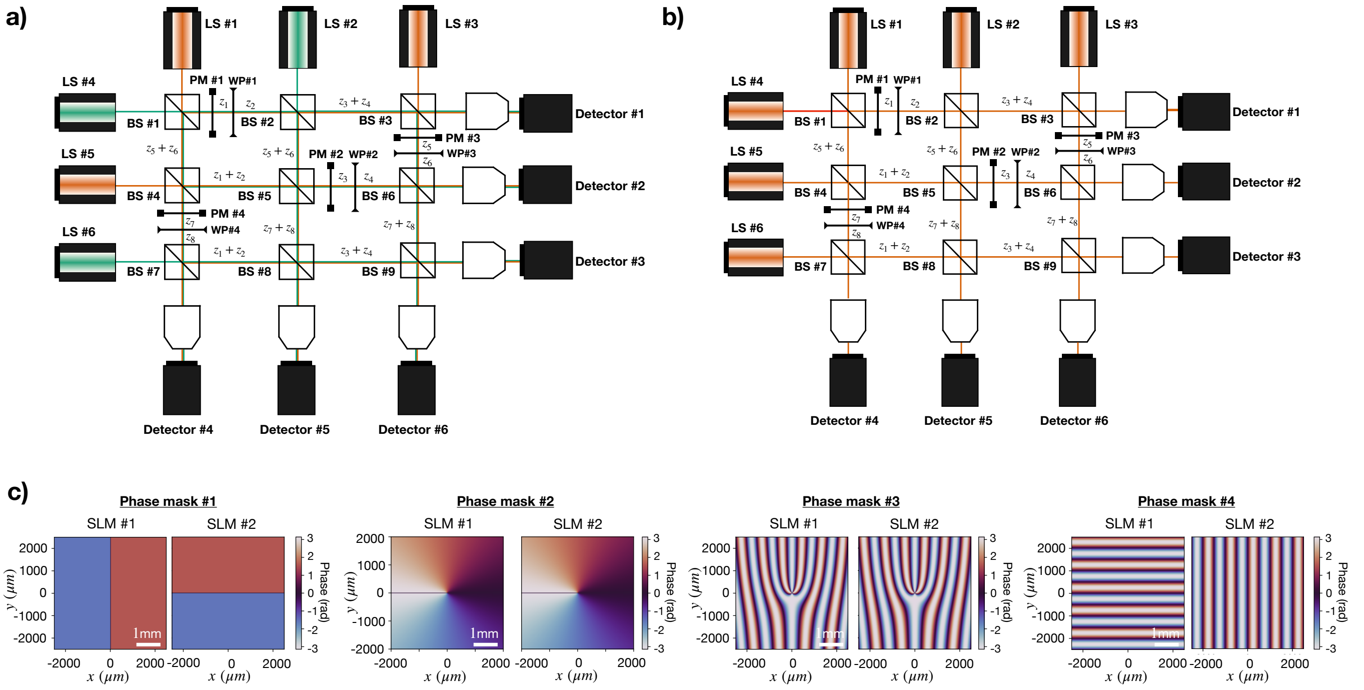

Supplementary Fig. 6. Topological discovery within a large-scale optical setup. The optical elements corresponding to phase masks (PM#1 - PM#4) remain fixed during the optimization. The parameter space (25 optical parameters) is defined by nine beam splitter ratios (BS#1 - BS#9), eight distances (z_1 - z_8) and four wave plates with variable applied retardance and orientation). After interacting with a high NA objective lens, light gets detected across six detectors (#1 - #6) with $0.05\mu m$ pixel size screen. The parameter update is driven by the camera demonstrating the minimum loss value. (a) Initial virtual setup for STED microscopy (Hell and Wichmann, 1994). The light sources emit 632.8 nm and 530 nm wavelength Gaussian beams that are linearly polarized at 45°. (b) Initial virtual optical setup for Dorn, Quabis and Leuchs (2003). It consists of six light sources emitting a 632.8 nm wavelength Gaussian beam that are linearly polarized at 45°. (c) Fixed phase patterns corresponding to each phase mask placed on super-SLMs (from #1 to #4). The phase mask #1 corresponds to the radial phase pattern originally used in STED microscopy. Phase mask #2 is the polarization converter demonstrated in Dorn, Quabis, and Leuchs (2003). Phase mask #3 corresponds to a forked grating of $p = 2$. Phase mask #4 correspond to horizontal and vertical gratings.

depicted in Supplementary Fig. 7. The optical system, depicted in Supplementary Fig. 7a, consists of 12 light sources emitting a 635 nm wavelength Gaussian beam that are linearly polarized at 45° interacting with 36 beam splitters and four phase masks displaying the fixed phase patterns in Supplementary Fig. 7b. The loss function corresponds to Supplementary Equation (6) considering the intensity of the electromagnetic field's longitudinal component, $|E_z|^2$, and $\epsilon = 0.7$. We set-up the AdamW optimizer with a step size of 0.05 and a weight decay of 10^{-4}. The system is initialized with random optical parameters with values between 0 and 1. The optimization is terminated if there is no improvement in the loss value over 500 consecutive iteration steps. This condition is checked every 100 steps. The system converged (in roughly 1 hour using a GPU) into the topology highlighted in Supplementary Fig. 7c., demonstrating the smallest loss value in the detector #3. The identified solution displays similar spot size as the reference (see Supplementary Fig. 7d).

D. Rediscovery through exploration in highly parameterized systems

We further task XLuminA to discover experimental setup topologies within larger parameter spaces, this time enabling the system to optimize the SLMs masks instead of having fixed phase patterns. The goal here is to discover both the optical topology using the available optical parameters (i.e., distances, beam splitter ratios and wave plate's angles) and the phase patterns to imprint onto the light beams (i.e., using SLMs).

For this purpose we build the 3×3 optical setup depicted in Supplementary Fig. 8. It consists of six light sources emitting linearly polarized Gaussian beams of wavelengths 650 nm and 532 nm. Three building blocks, which contain

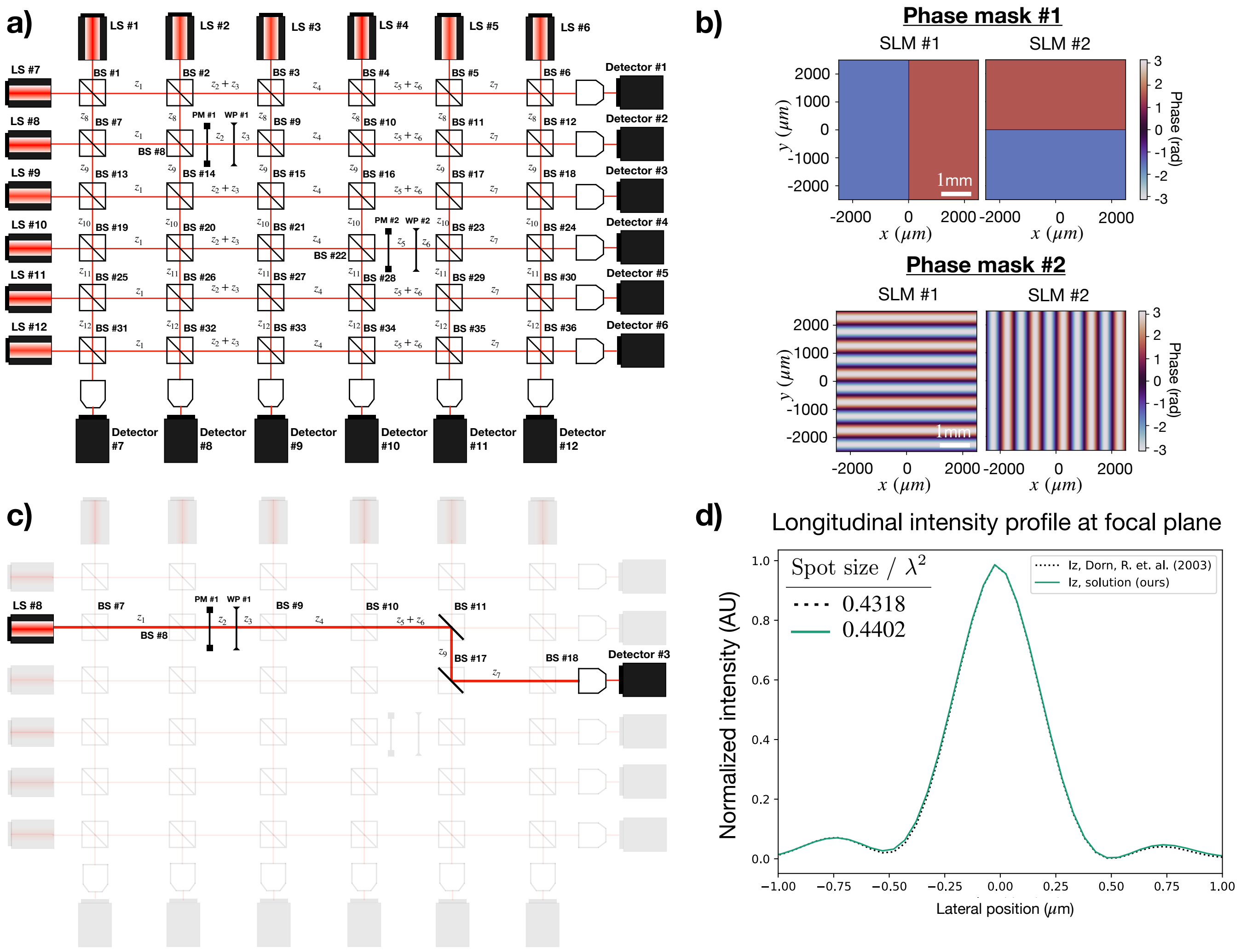

Supplementary Fig. 7. Pure topological discovery within a 6 × 6 large-scale optical setup. The optical elements corresponding to phase masks (PM#1 and PM#2) remain fixed during the optimization. The parameter space is defined by 36 beam splitter ratios (BS#1 - BS#36), 12 distances (z_1 - z_{12}) and 2 wave plates with variable applied retardance and orientation). After interacting with a high NA objective lens, light gets detected across 12 detectors (#1 - #6) with 0.05μm pixel size screen. The parameter update is driven by the camera demonstrating the minimum loss value. (a) Initial virtual setup for Dorn, Quabis and Leuchs (2003). It consists of 12 light sources emitting a 635 nm wavelength Gaussian beam that are linearly polarized at 45°. (b) Fixed phase patterns corresponding to each phase mask placed on super-SLMs. The phase mask #1 corresponds to the polarization converter demonstrated in Dorn, Quabis, and Leuchs (2003). Phase mask #2 correspond to horizontal and vertical gratings. (c) Discovered topology. The minimum value of the loss is demonstrated in detector #3. The beam splitter ratios, in [Transmittance, Reflectance] pairs correspond to: BS#7 [0.999, 0.001], BS#8 [0.996, 0.004], BS#9 [0.841, 0.159], BS#10 [0.791, 0.209], BS#11 [0.015, 0.984], BS#17 [0.280, 0.720], and BS#18 [0.962, 0.038]. Wave plate's (in radians) η = 3.13 and θ = 3.13. Distances (in cm) correspond to z_1 = 167.15, z_2 = 133.78, z_3 = 102.45, z_4 = 65.06, z_5 = 81.97, z_6 = 71.99, z_7 = 104.96 and z_9 = 169.96. (d) Normalized longitudinal intensity profile, $|E_z|^2$, for Dorn, Quabis, and Leuchs (2003) and the identified solution (black dotted, and green lines, respectively). Lateral position indicates lateral distance from the optical axis. The spot size is computed as $\phi = (\pi/4)\text{FWHM}_x\text{FWHM}_y$, where FWHM denotes for Full Width Half Maximum.

one super-SLM (i.e., two SLMs imprinting independent phase masks to orthogonal polarization states) and a wave plate separated a distance z, are placed within the diagonal of the grid (grey boxes in Supplementary Fig. 8). Light gets ultimately detected across six detectors. As discussed in the previous section, the loss function is computed at each detector, the parameter update is driven by the device demonstrating the minimum value. This selection is conducted in a fully differentiable manner using `jax.nn.logsumexp()`.

We first target XLuminA to rediscover the concept of STED microscopy within the general setup in Supplementary Fig. 8. The parameter space ($\sim$ 4 million parameters) corresponds to three super-SLMs (i.e., 6 SLMs) with a resolution

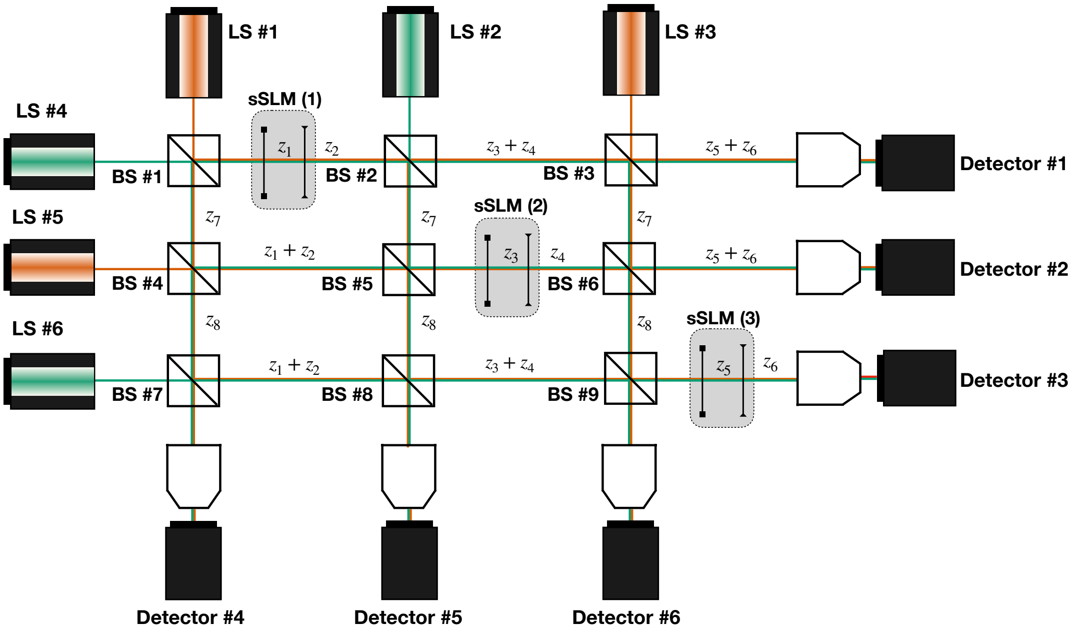

Supplementary Fig. 8. Virtual optical setup utilized for large-scale discovery. It features light sources that emit Gaussian beams with wavelengths of 650 nm and 532 nm linearly polarized at 45°. Gray boxes, numbered from (1) to (3), represent the building units, each comprising one super-SLM (sSLM) and one wave plate (WP). Distances are denoted as z_i where $i = 1, ..., 8$. After interacting with a high NA objective lens (of NA= 0.9), light gets detected across six detectors (#1 - #6) with $0.05\mu m$ pixel size screen. The parameter space ($\sim$ 4 million optical parameters) contains three sSLM, four wave plates (WPs) with variable phase retardance η and orientation angle θ, eight distances and nine beam splitter ratios. To discover the experimental setup topologies for Dorn, Quabis and Leuchs (2003): (1) all the light sources are set to emit 635 nm wavelength, (2) the resolution is set to 1024×1024 pixels with a pixel size of 4.8 μm and (3) the parameter space is of $\sim$ 6.3 million optical parameters (containing three sSLM, four WPs with variable phase retardance η and orientation angle θ, eight distances and nine beam splitter ratios).

of 824×824 pixels with a pixel size of $6.06\mu m$, three wave plates, eight distances and nine beam splitter ratios. The loss function corresponds to Supplementary Equation (6), in this instance considering the radial intensity of the effective light emerging from the STED process, $|E_x|^2 + |E_y|^2$, and $\varepsilon = 0.5$. We simulate the stimulated emission depletion effect using Supplementary Equation (1) with the efficiency set to $\beta = 1$. We set-up the AdamW optimizer with a step size of 10^{-3} and a weight decay of 10^{-2}. The system is initialized with random optical parameters with values between 0 and 1. The optimization is terminated if there is no improvement in the loss value over 500 consecutive iteration steps. This condition is checked every 100 steps. The loss value evolution over the number of iteration steps is depicted in Supplementary Fig. 9d. The system converged (in roughly 1.3 hours using a GPU) into the topology highlighted in Fig. 5a of the main manuscript, demonstrating the smallest loss value in the detector #2. The discovered topology can be identified from the beam splitter ratios starting from detector #2: $BS\#6$ has 99.9% transmittance, which implies that light is directly is coming from $BS\#5$. In turn, $BS\#5$ has a reflectance of 99.9% which redirects light from $BS\#2$, which has a transmittance of 79.9%. This already defines light source #2 as the emitter of the green (excitation) beam. Then, the remaining light comes from $BS\#1$ which shows a 99.9% reflectance, meaning that the red light comes from source #1.

To rediscover the concept used in Dorn, Quabis and Leuchs [12], we initialize the system using the general setup in Supplementary Fig. 8. This time, however, the light sources emitting 532 nm wavelength are switched to emit 650 nm. The parameter space ($\sim$ 6.2 million parameters) corresponds to three super-SLMs (i.e., 6 SLMs) with a resolution of 1024×1024 pixels with a pixel size of $4.8\mu m$, three wave plates, eight distances and nine beam splitter ratios. The loss function corresponds to Supplementary Equation (6), in this instance considering the intensity from the longitudinal component of the electric field, $|E_z|^2$, and $\varepsilon = 0.7$. We set-up the AdamW optimizer with a step size of 0.05 and a weight decay of 10^{-5}. The system is initialized with random optical parameters with values between 0 and 1. The optimization is terminated if there is no improvement in the loss value over 500 consecutive iteration steps. This condition is checked every 100 steps. The loss value evolution over the number of iteration steps is depicted in Supplementary Fig. 9e. The system converged (in roughly 35 minutes using a GPU) into the topology highlighted

in Fig. 6a, demonstrating the smallest loss value in the detector #6. As for the previous examples, the discovered topology can be identified from the beam splitter ratios: starting from detector #6, $BS\#9$ has 99.9% transmittance, which implies that light is directly is coming from $BS\#6$. In turn, $BS\#6$ has a reflectance of 99.9%. This means that light has interacted with $BS\#5$ and gone through the sSLM and WP. Then, $BS\#5$ has has a reflectance of 99.9%, which defines the incoming light at $BS\#2$. This, in turn, shows a 99.9% transmittance, meaning that light comes from the light source #2.

E. Discovery of a previously unreported experimental blueprint

Finally, we demonstrate the capabilities of XLuminA for genuine discovery. We use the same initial optical setup in Supplementary Fig. 8. The parameter space ($\sim$ 4 million parameters) corresponds to three super-SLMs (i.e., 6 SLMs) with a resolution of 824×824 pixels with a pixel size of $6.06 \mu m$, three wave plates, eight distances and nine beam splitter ratios. The loss function corresponds to Supplementary Equation (6), in this instance considering the total intensity of the effective light emerging from the STED process, $|E_x|^2 + |E_y|^2 + |E_z|^2$, and $\varepsilon = 0.5$. We simulate the stimulated emission depletion effect using Supplementary Equation (1) with the efficiency set to $\beta = 1$. We set-up the AdamW optimizer with a step size of 10^{-3} and a weight decay of 10^{-3} and initialize the system with random optical parameters with values between 0 and 1. The optimization is terminated if there is no improvement in the loss value over 500 consecutive iteration steps. This condition is checked every 100 steps. The loss value evolution over the number of iteration steps is depicted in Supplementary Fig. 9f. The system converged (in roughly 3.8 hours using a GPU) into the topology highlighted in Fig. 7a, demonstrating the smallest loss value in the detector #2. The discovered topology can be identified from the beam splitter ratios starting from detector #2: $BS\#6$ has 99.9% transmittance, which implies that light is directly is coming from $BS\#5$. In turn, $BS\#5$ has a reflectance of 99.9% which redirects light from $BS\#2$, which has a transmittance of 66.2%. This already defines light source #2 as the emitter of the green (excitation) beam. Then, the remaining light comes from $BS\#1$ which shows a 99.9% reflectance, meaning that the red light (depletion) comes from source #1.

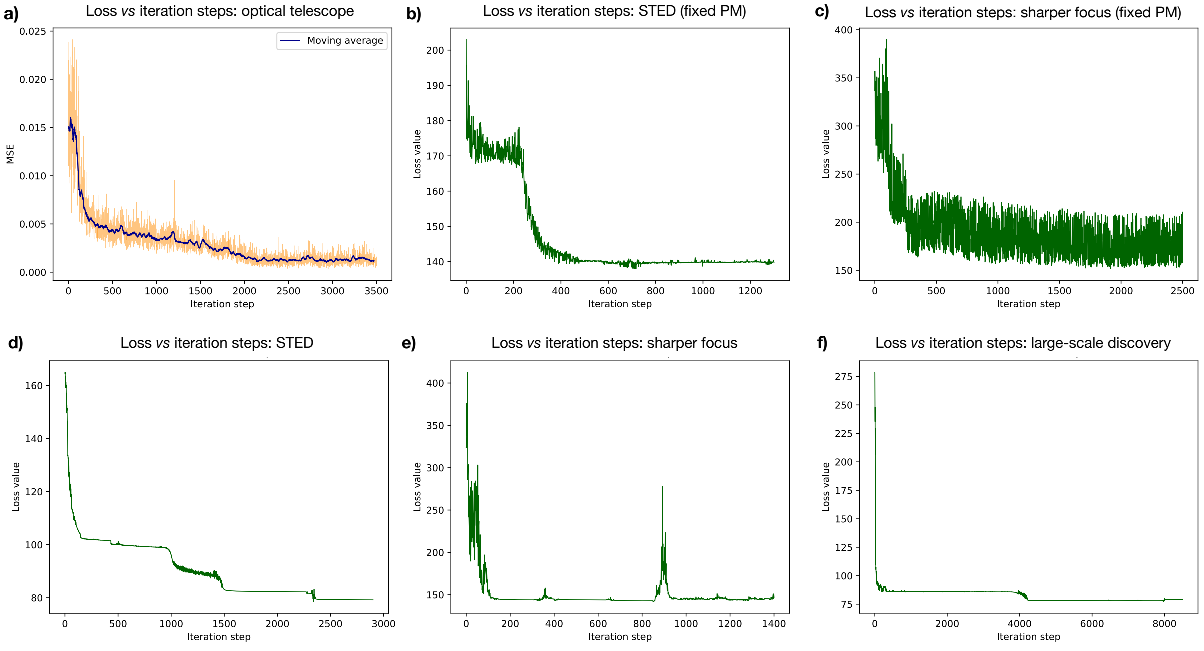

Supplementary Fig. 9. Loss value over iteration steps for the different optical experiments shown in this work. (a) The optical system used to adjust beam and image sizes (i.e., an optical telescope). This solution was identified in roughly 2.4 hours using a GPU. This time includes optics simulations and the data loading processes. (b) The virtual optical setup with fixed phase masks (PM) for the sharper focus employing optical vortices. This solution was identified in roughly 35 minutes using a GPU. (c) The virtual optical setup with fixed phase masks (PM) for STED microscopy. This solution was identified in roughly 1 hour using a GPU. (d) The virtual optical setup for STED microscopy. This solution was identified in roughly 1.3 hours using a GPU. (e) The virtual optical setup for the sharper focus employing optical vortices. This solution was identified in roughly 35 minutes using a GPU. (f) The previously unreported experimental blueprint. This solution was identified in roughly 3.8 hours using a GPU.

[1] F. Shen and A. Wang, Fast-fourier-transform based numerical integration method for the rayleigh-sommerfeld diffraction formula, Applied Optics **45**, 1102 (2006).
[2] H. Ye, C.-W. Qiu, K. Huang, J. Teng, B. Luk'Yanchuk, and S. Yeo, Creation of a longitudinally polarized subwavelength hotspot with an ultra-thin planar lens: Vectorial rayleigh-sommerfeld method, Laser Physics Letters **10** (2013).
[3] Y. Hu, Z. Wang, X. Wang, *et al.*, Efficient full-path optical calculation of scalar and vector diffraction using the bluestein method, Light: Science & Applications **9**, 119 (2020).
[4] J. Li, Z. Fan, and Y. Fu, FFT calculation for Fresnel diffraction and energy conservation criterion of sampling quality, in *Lasers in Material Processing and Manufacturing*, Vol. 4915 (SPIE, 2002) pp. 180 – 186.
[5] L. M. S. Brea, Diffractio, python module for diffraction and interference optics (2019).
[6] J. Nocedal and S. J. Wright, *Numerical optimization* (Springer).
[7] P. Virtanen, R. Gommers, T. E. Oliphant, M. Haberland, T. Reddy, D. Cournapeau, E. Burovski, P. Peterson, W. Weckesser, J. Bright, S. J. van der Walt, M. Brett, J. Wilson, K. J. Millman, N. Mayorov, A. R. J. Nelson, E. Jones, R. Kern, E. Larson, C. J. Carey, İ. Polat, Y. Feng, E. W. Moore, J. VanderPlas, D. Laxalde, J. Perktold, R. Cimrman, I. Henriksen, E. A. Quintero, C. R. Harris, A. M. Archibald, A. H. Ribeiro, F. Pedregosa, P. van Mulbregt, and SciPy 1.0 Contributors, SciPy 1.0: Fundamental Algorithms for Scientific Computing in Python, Nature Methods **17**, 261 (2020).
[8] D. P. Kingma and J. Ba, Adam: A method for stochastic optimization (2017), arXiv:1412.6980 [cs.LG].
[9] S. W. Hell and J. Wichmann, Breaking the diffraction resolution limit by stimulated emission: stimulated-emission-depletion fluorescence microscopy, Optics Letters **19**, 780 (1994).
[10] M. Hofmann, C. Eggeling, S. Jakobs, and S. W. Hell, Breaking the diffraction barrier in fluorescence microscopy at low light intensities by using reversibly photoswitchable proteins, Proceedings of the National Academy of Sciences **102**, 17565 (2005).
[11] T. G. Mayerhöfer, S. Pahlow, and J. Popp, The bouguer-beer-lambert law: Shining light on the obscure, Chemphyschem **21**, 2029 (2020).
[12] R. Dorn, S. Quabis, and G. Leuchs, Sharper focus for a radially polarized light beam, Physical Review Letters **91**, 233901 (2003).
